# Genetic Variations of ****α****-Methylacyl-CoA Racemase Are Associated with Sporadic Prostate Cancer Risk in Ethnically Homogenous Koreans

**DOI:** 10.1155/2013/394285

**Published:** 2013-12-07

**Authors:** Sang-Jin Lee, Jae Young Joung, Hyekyoung Yoon, Jeong Eun Kim, Weon Seo Park, Ho Kyung Seo, Jinsoo Chung, Jung-Ah Hwang, Seung-Hyun Hong, Seungyoon Nam, Sohee Park, Jeongseon Kim, Kang Hyun Lee, Yeon-Su Lee

**Affiliations:** ^1^Genitourinary Cancer Branch, National Cancer Center, Goyang 410-769, Republic of Korea; ^2^Center for Prostate Cancer, National Cancer Center, 111 Jungbalsan-ro, Ilsandong-gu, Goyang, Gyeonggi-do 410-769, Republic of Korea; ^3^Cancer Biostatistics Branch, National Cancer Center, Goyang 410-769, Republic of Korea; ^4^Cancer Genomics Branch, National Cancer Center, 111 Jungbalsan-ro, Ilsandong-gu, Goyang, Gyeonggi-do 410-769, Republic of Korea; ^5^Molecular Epidemiology Branch, National Cancer Center, Goyang 410-769, Republic of Korea; ^6^Department of Biostatistics, Graduate School of Public Health, Yonsei University, Seoul 120-752, Republic of Korea

## Abstract

*Background*. To assess if the variants of (R)-alpha-methyl-CoA racemase (AMACR) gene would be associated with the risk of sporadic prostate cancer in ethnically homogenous Koreans. *Materials and Methods*. We enrolled 194 patients with prostate cancer and 169 healthy controls. A total of 17 single nucleotide polymorphisms of the AMACR gene were selected. The distribution of each genotype and haplotype was analyzed and their association with the incidence of prostate cancer was evaluated. Further, we detected AMACR expression in tumor with immunohistochemistry and analyzed its association with genotype regarding prostate cancer risk. *Results*. AG or GG genotype of rs2278008 (E277K) tended to lower prostate cancer risk. The minor G allele was found to be a significant allele that decreased the risk of prostate cancer (adjusted OR, 0.57; 95% CI, 0.35–0.93, *P* value = 0.025). In patients expression AMACR, AG or GG genotype was also significant genotype in terms of prostate cancer risk (adjusted OR, 0.47; 95% CI, 0.26–0.87, *P* value = 0.017). Further, [GGCGG] haplotype consisted of five coding SNPs of rs2278008, rs34677, rs2287939, rs10941112, and rs3195676 which decreased the risk of prostate cancer (*P* value = 0.047). *Conclusions*. Genetic variations of AMACR are associated with the risk of sporadic prostate cancer that underwent radical prostatectomy in Koreans.

## 1. Background

The detailed etiology of prostate cancer is still unclear; it is a very heterogeneous disease due to the involvement of various inherited genetic elements and environment factors, including a fatty diet. Many studies have sought to identify the risk factors of prostate cancer, mainly by a targeted gene approach, which has confirmed the effect of known carcinogenesis genes [1–4]. Recently, many studies including genome-wide association studies (GWAS) identified significant associations between lots of single nucleotide polymorphisms (SNPs) and prostate cancer [5–9]. Some studies identified chromosome 5p13, the site of the gene encoding (R)-alpha-methyl-CoA racemase (AMACR), as the location of a prostate cancer susceptibility gene [10–12].

AMACR is catalytically involved in fatty acid oxidation, which converts (R)-alpha-methyl-branched-chain fatty acyl-CoA ester to the (S)-stereoisomer. AMACR is critical in prostate cancer cell progression; the downregulation of AMACR expression hampers the proliferation of the LAPC-4 androgen-responsive prostate cancer cells [13]. AMACR is abundantly expressed and is recognized as a standard tissue biomarker capable of a highly sensitive and specific diagnosis of prostate cancer [14–16]. An AMACR spliced variant was reported capable of creating a novel transcript that is expressed with other forms of AMACR in prostate cancer [17]. These findings point to the value of AMACR and its variants in developing diagnostic biomarkers that will complement the diagnostic capability of PSA, while addressing the limitations of PSA, specifically its low specificity. The chromosomal region of AMACR has been validated as a susceptible locus for prostate cancer, including hereditary prostate cancer [10, 11]. Studies addressing the potential linkage between AMACR polymorphisms and sporadic prostate cancer risk in different populations, however, have not been consistent and have precluded any definitive conclusion [18–20]. The genetic heterogeneity of ethnically different study populations might have lead to these inconsistent results. This study was designed to assess whether genetic variations of AMACR were associated with sporadic prostate cancer development in Korean men, known to be an ethnically homogenous population [21], by investigating the impact of AMACR polymorphisms on the risk of prostate cancer and clinical features.

## 2. Materials and Methods

### 2.1. Study Subjects

The investigation was a hospital-based case-control study of prostate cancer. Both prostate cancer patients and controls were of the same Korean ethnic origin with all residents born in Korea. Any subject who had any relative with a past history of prostate cancer among their first-degree relatives was excluded. Patients who were histologically confirmed to have prostate cancer were enrolled at the National Cancer Center in Korea between January 2005 and February 2009. Controls were confirmed to be free of prostate cancer by determination of the blood PSA level and by digital rectal examination. Although the PSA level of three controls continually increased and exceeded 4 ng/mL, prostate needle biopsy conducted in the three subjects confirmed the absence of prostate cancer. All patients underwent radical prostatectomy and Gleason grading was determined with prostatectomy specimens. Demographic and clinical data were based on prostate cancer database in our institution. We obtained the written informed consent for participation in the study from all participants. This study was approved by the Institutional Review Board of the National Cancer Center in Korea (NCCNCS05-049).

### 2.2. Single Nucleotide Polymorphism (SNP) Selection and Genotyping

The target SNPs of AMACR were selected in the National Center for Biotechnology Information (NCBI) database version (http://www.ncbi.nlm.nih.gov/projects/SNP/). We collected the information of all coding SNPs in AMACR with heterozygosity > 0; a total of 17 SNPs were identified (see Supplemental Table 1 available online at http://dx.doi.org/10.1155/2013/394285). From these, four SNPs with a heterozygosity of 0.5 were excluded. Additional two SNPs (rs76184600 and rs117220551) were also excluded because of assay design failure to create suitable primers. Finally, primers for 11 SNPs were designed using DESINGER (Sequenom, CA, USA) software (Supplemental Table 2). Genomic DNA was prepared from 200 *μ*L of peripheral blood using the QIAamp Blood Kit (QIAGEN, Valencia, CA, USA) according to the manufacturer's instructions. Genotyping was performed by the previously described method [1] and the resulting genotype data were obtained by Typer (version 4.0, Sequenom) and were subjected to statistical analysis.

### 2.3. AMACR Expression in Prostate Cancer

At least three core tissue biopsies (each 3 mm in diameter) were taken from morphologically representative regions of each paraffin embedded prostate tumor and precisely arrayed using a custom built instrument. To identify AMACR protein expression, 4 *μ*m thick tissue sections were placed on glass slides for histological examination, one slide for staining with hematoxylin/eosin and the other slide for immunostaining with anti-AMACR antibody. On the basis of histological analysis, expression of AMACR protein in each patient was determined in a blinded fashion by a single experienced pathologist. Slides were scored by a semiquantitative method. The intensity of AMACR expression was graded on a score of 0 to 3+ (0 = no staining, 1+ = weakly positive, 2+ = positive, and 3+ = strongly positive).

### 2.4. Statistical Analyses

The demographic and clinical characteristics in the study population and controls were analyzed using a chi-square test for categorical variables and an independent *t*-test for continuous variables. Hardy-Weinberg equilibrium (HWE) was determined separately in cases and controls for each SNP. SNPs that deviated from the HWE in the control group were excluded (*χ* ^2^ test at *P* value <0.05). The association between AMACR polymorphisms and prostate cancer risk was evaluated on the basis of genotypic frequencies of each SNP using an unconditional logistic regression model. The Cochran-Armitage trend test was also used to analyze the linear trend, as expressed by odds ratios (ORs). The haplotype estimation was carried out using the PHASE program (version 2.1; http://stephenslab.uchicago.edu/software.html#phase), which implements a Bayesian statistical method for reconstructing haplotypes. An unconditional logistic regression determined the association between AMACR haplotypes and prostate cancer risk, considering the haplotypic frequencies of each haplotype and the haplotype pair of each person. Additionally, the associations between genotypes and clinical parameters were determined using an unconditional logistic regression. To assess the power for detecting association, we used the power for genetic association analyses (PGA) [22]. All other statistical analyses were performed using SAS (version 9.1 SAS Institute, NC, USA). The reported *P* values are two-sided, and a significance level < 5% was considered statistically significant.

## 3. Results

### 3.1. Subjects Characteristics

Demographic and clinical characteristics of 194 prostate cancer patients and 169 controls are summarized in Table 1. Statistically significant differences between cases and controls were evident with respect to age, serum PSA level, and drinking status. Most of the prostate cancer patients displayed a high serum PSA level, with the risk increasing with age, as expected. No significant differences were found in smoking status, body mass index, or hypertension (Table 1).

### 3.2. AMACR Polymorphisms and Prostate Cancer Risk

Genotype frequencies for all of the target SNPs of AMACR in this study are presented in Supplemental Table 3. From 11 cSNPs, one SNP failed during the data quality control process because of high primer-dimer potential. Five cSNPs were revealed to be monomorphic for the Korean subjects. For the remaining five cSNPs (rs2278008, rs34677, rs2287939, rs10941112, and rs3195676), the association with prostate cancer was analyzed and is summarized in Table 2. One missense SNP (Glu277Lys), rs2278008, was found to be associated with prostate cancer risk. Individuals with the AG or GG genotype of rs2278008 revealed to have a lower risk compared to those with the AA genotype (adjusted OR, 0.51; 95% CI, 0.29–0.89, *P* = 0.018). Also, comparison of the allele frequencies showed a statistically significant higher frequency of G allele in the control group than the case group, indicating that the G allele was associated with a lower risk of prostate cancer than the A allele (adjusted OR, 0.57: 95% CI, 0.35–0.93, *P* = 0.025). One other cSNP (G175D), rs10941112, also showed a significant association for the GA genotype which was evident by an increased prostate cancer risk (OR, 1.60; 95% CI, 1.02–2.52, *P* = 0.041), although it was no more significant after adjustment. No significant evidence of prostate cancer association with the remaining SNPs was identified.

### 3.3. Association of the AMACR Polymorphism with Clinical Factors of Prostate Cancer

Reclassifying the patients into two groups according to their GS produced 95 patients (49.0%) with GS ≥ 7 and 99 patients (51.0%) with GS < 7. Interestingly, for patients with GS ≥ 7, the GA or AA genotype of rs10941112 and rs3195676 showed statistically significant association with increased risk of prostate cancer with an adjusted OR of 2.28 (*P* = 0.010) and 2.35 (*P* = 0.008), respectively, (Table 3). The same genotypes of these cSNPs were also associated with an increased risk of prostate cancer with statistical significance for the patients with pTstage ≥ pT2c (adjusted OR 2.60, *P* = 0.007 for rs10941112 and an adjusted OR of 2.66, *P* = 0.006 for rs3195676).

### 3.4. AMACR Polymorphisms and Risk of Prostate Cancer with Amacr Expression

For the case group, AMACR expression in tumor tissues was determined by immunostaining and reclassified into two subgroups according to expression level: a negative group (score 0) versus a positive group (score 1+, 2+ or 3+). There were 39 cases (20.2%) in the negative group and 154 cases (79.8%) in the positive group (Table 1). Between variants of AMACR and intensity of AMACR expression, no significant relation was found except for rs2287939 (Supplemental Table 4). In the case group expressing AMACR protein, individuals with the AG or GG genotype of rs2278008 showed a decreased prostate cancer risk compared to those with the AA genotype (adjusted OR 0.47; 95% CI, 0.26–0.87, *P* = 0.017; Table 4 and supplemental figure).

Considering the clinical features, the AG or GG genotype of rs2278008 showed a significantly decreased risk of prostate cancer with GS ≥ 7 (adjusted OR 0.43; 95% CI, 0.19–0.99, *P* = 0.048). Additionally, the GT or TT genotype of rs34677 was associated with a statistically significant decreased risk for patients with ≥pT2c (adjusted OR 0.35; 95% CI, 0.15–0.79, *P* = 0.012). Also, the patients with GA or AA genotype of rs10941112 and rs3195676 revealed to have a significantly increased risk of developing unfavorable prostate cancer with ≥pT2c (Table 5).

### 3.5. Haplotype Analysis of AMACR Polymorphism

The whole frequency distribution and association analysis of haplotypes for SNPs in AMACR are summarized in Table 6 (in Supplemental Table 5). When the haplotypes consisted of all five cSNPs, individuals with the 12345-5 [GGCGG] haplotype showed a significantly lower risk of prostate cancer compared to the subjects with the most frequent haplotype 12345-1 [AGCAA] (adjusted OR, 0.41; 95% CI, 0.17–1.00, *P* = 0.049). Furthermore, individuals with one or more copies of the [GGCGG] tended to have a significantly decreased risk of prostate cancer compared to those without [GGCGG] haplotype (adjusted OR, 0.41; 95% CI, 0.17–0.99, *P* = 0.047). Interestingly, when we considered the haplotype pair of each individual, the haplotype pair harboring the G allele of SNP rs2278008 revealed a lowered risk in combination with the G allele of rs34677, rs1094112, and rs3195676 (Table 6).

## 4. Discussion

AMACR is a key enzyme in the ß-oxidation catabolic pathway of fatty acids and is known to be upregulated in several cancers including prostate cancer [23–26]. Sequence variants of AMACR have been previously investigated to find their association with prostate cancer risk [10–12, 27, 28]. Results, however, were inconsistent: One study suggested that variants in AMACR gene were associated with familial, but not sporadic, prostate cancer [10] and other subsequent studies reported no association between sporadic prostate cancer and AMACR gene variants [18, 19] (http://dceg.cancer.gov/research/how-we-study/genomic-studies/cgems-summary/). In the subgroup analysis of one of these studies, a tendency toward decreased risk in homozygous white carriers of the variant alleles for both rs3195676 and rs10941112 was evident [19]. Thus, genetic studies about the association of genetic polymorphisms with prostate cancer risk might show different results depending on the ethnic background of the subjects. The use of Korean men, who are reported as an ethnically homogenous population [21], may deliver useful conclusion for the association of AMACR polymorphisms with prostate cancer risk, by reducing the possible influences related to genetic heterogeneity. Here, we found statistically significant association of AMACR polymorphisms with prostate cancer risk using single ethnic Koreans without any family history of immigration from other countries. Further, we analyzed the risk of prostate cancer in relation with AMACR polymorphism in cases of AMACR expression.

In our study, rs2278008 (E277K) was associated with prostate cancer risk in ethnically homogenous Koreans with statistical significance. To our knowledge, this is the first report of a protective effect of rs2278008 of the AMACR gene on the development of sporadic prostate cancer. Although, rs2278008 SNP of AMACR was reported previously with the significantly different genotype frequencies between hereditary prostate cancer groups and controls, this tendency was not shown when compared with a sporadic prostate cancer group [10]. In our study, the GG or AG genotype of rs2278008 was more frequently observed in the control group when compared with the sporadic prostate cancer group. Furthermore, in subjects expressing AMACR, the association of rs2278008 with prostate cancer risk also showed statistical significance. With respect to the association of the gene variant with clinical factors of prostate cancer, recent data as part of the Michigan Prostate Cancer Genetics Project demonstrated a significant linkage between markers on 5p13-q11 and prostate cancer aggressiveness, as defined by GS [29]. These observations suggest that this region of the genome may harbor sequence variations associated with prostate cancer risk and the extent of tumor differentiation may be considered as a predictor for prognosis. We also found genetic variations of AMACR related to the histologic grade of prostate cancer. Patients with a specific sequence variant of rs2278008 and rs34677 tended to show a decreased risk of developing unfavorable prostate cancer with GS ≥ 7 or ≥pT2c. On the contrary, patients with the GA or AA genotype of rs10941112 and rs3195676 displayed a significantly increased risk of developing unfavorable prostate cancer. Based on this result, the genetic polymorphism of AMACR might influence the development of prostate cancer for patients expressing AMACR. Additionally, haplotype analysis revealed the combinatorial impact of individual SNPs clearly. The protective effect of rs2278008 may strongly influence cancer susceptibility with the risk decreased by the effect of rs34677, rs10941112, or rs3195676, since the G allele of rs2278008 was mostly revealed as statistically significant and the protective effects in any combination with G allele of rs34677, rs10941112, or rs3195676, but protective effects were not present with the others. This is the first report ever for the analysis of the combinatorial effect of AMACR SNPs on the prostate cancer susceptibility in haplotype.

Unlike other cancers, prostate cancer cells use fatty acid as their main energy source instead of glucose [30–32]. AMACR is required to enter into the fatty acid oxidation, resulting in energy production for the cancer cells [30, 31]. Combining our discovery (the protective role of rs2278008 in AMACR), the missense mutation by the minor allele of the sequence variant might disrupt or decrease the enzyme (AMACR) activity, subsequently leading to unfavorable energy supply in the cancer cells. The speculation can explain the lower prostate cancer risk for the missense mutation-harboring patients, which needs to be further studied. In addition, three-dimensional structural data implicate that the substitution encoded by rs1094112 (G175D) may directly impact the stability of the AMACR protein backbone [33]. Otherwise, no studies have investigated the impact of genetic variations on characteristics of prostate cancer by changing functional structure.

Despite the new findings that the sequence variants of AMACR are related to the risk of sporadic prostate cancer in the ethnically homogenous population of Korean men, caution should be taken when interpreting our findings due to some limitations. Firstly, because of the relatively small number of cases and controls in this study, the power of this study is limited. Particularly, stratified analyses with respect to clinical factors of prostate cancer might be underpowered. By using PGA method, the SNPs in our study were were calculated about 5%~ about 43% power at an *σ* value of 5%, although it has low power, and statistically significant finding was detected. Instead of each value for statistical power, we just presented wide range of statistical power because of many subgroups including clinical factors of prostate cancer. Secondly, the patients were older and consumed less alcohol than the controls, and the number of patients having a family history of prostate cancer (not first-degree relatives) was more than the controls, although it was not statistically significant. To minimize the effect of the different distribution in cases and controls, we adjusted the age, smoking quantity, alcohol drinking, and family history of prostate cancer in statistical analysis. Furthermore, our primary interest lay in the genotype and its association with prostate cancer risk, and it is unlikely that the genotype is affected by age. Thus, we believe that the bias possibly caused by different age distributions among cases and controls would minimally influence for the results of our study.

## 5. Conclusions

We concluded that the genetic variations of AMACR were associated with the risk of sporadic prostate cancer treated with radical prostatectomy in ethnically homogenous population of Korean men. Our investigation of the relationship between genotype frequencies, haplotype pair, and the expression of AMACR protein in tumors with clinical features demonstrates that sequence variants of AMACR may play an important role in the development of prostate cancer.

## Supplementary Material

We collected the information of all coding SNPs in AMACR with heterozygosity > 0; a total of 17 SNPs were identified (Supplemental Table 1). Primers for 11 SNPs were designed using DESINGER (Sequenom, CA, USA) software (Supplemental Table 2). Genotype frequencies for all of the target SNPs of AMACR in this study are presented in Supplemental Table 3. Supplemental Table 4 shows the relation between variants of AMACR and intensity of AMACR expression. Supplemental Table 5 represents the AMACR (5p13) haplotypes and their association with prostate cancer risk. Supplemental figure describes results of immunohistochemical staining of AMACR according to variant genotypes of rs2278008.

## Figures and Tables

**Table 1:** Demographic and clinical characteristics of the subjects.

Variables	Cases (*n* = 194)		Controls (*n* = 169)		*P* value^a^
Age, years (median)	66.52 ± 7.16 (68.0)		60.13 ± 3.30 (61.0)		<0.001
Serum PSA, ng/mL (median)	21.28 ± 28.15 (9.8)		1.47 ± 1.63 (1.0)		<0.001
Serum PSA (ng/mL), no.					<0.001
<4	11 (5.7%)		160 (94.7%)		
4–10 (≥4 to <10)	87 (44.9%)		8 (4.7%)		
10–20 (≥10 to <20)	43 (22.2%)		1 (0.6%)		
≥20	53 (27.3%)		0 (0.0%)		
BMI, kg/m^2^ (median)	24.35 ± 2.60 (24.1)		24.42 ± 2.34 (24.2)		0.800
BMI, no.					0.388
<25	127 (65.5%)		102 (61.1%)		
≥25	67 (34.5%)		65 (38.9%)		
Smoking, pack years (median)	21.93 ± 21.75 (18.3)		19.24 ± 20.60 (14.9)		0.264
Smoking status, no.					0.672
Never	52 (26.8%)		42 (24.9%)		
Ever	142 (73.2%)		127 (75.2%)		
Drinking status, no.					0.002
Never	68 (35.1%)		34 (20.1%)		
Ever	126 (65.0%)		135 (79.9%)		
Hypertension, no.					0.502
No	120 (61.9%)		109 (65.3%)		
Yes	74 (38.1%)		58 (34.7%)		
Family history of prostate cancer^†^					0.132
No	186 (95.9%)		146 (98.6%)		
Yes	8 (4.1%)		2 (1.4%)		
Gleason score, no.					
2–6	99 (51.0%)				
7	62 (32.0%)				
8–10	33 (17.0%)				
pTstage, no.					
pT0	6 (3.1%)				
pT2a	37 (19.1%)				
pT2b	3 (1.6%)				
pT2c	89 (45.9%)				
pT3a	31 (16.0%)				
pT3b	28 (14.4%)				

Immunohistochemistry of AMACR	Serum PSA, ng/mL				*P* value^a^
	<4	4–10 (≥4 to <10)	10–20 (≥10 to <20)	≥20	

0 (no staining)	1 (2.6%)	10 (25.6%)	7 (18%)	21 (53.9%)	0.004
1+ (weakly positive)	0 (0%)	16 (47.1%)	9 (26.5%)	9 (26.5%)	
2+ (positive)	5 (9.1%)	25 (45.5%)	12 (21.8%)	13 (23.6%)	
3+ (strongly positive)	5 (7.7%)	36 (55.4%)	15 (23.1%)	9 (13.9%)	

PSA: prostate specific antigen; BMI: body mass index; no.: number; AMACR: (R)-alpha-methyl-CoA racemase.

^a^Pearson's *χ* ^2^ test for categorical variables and independent *t-*test for continuous variables; two-sided *P*-values.

^†^“Family” represents relatives with the exception of first-degree relative, excluding 21 persons with missing values in the control group.

**Table 2:** Frequency of AMACR (5p13) polymorphisms and association with prostate cancer risk.

SNP	Genotype	Cases (%)	Controls (%)	Crude OR (95 %CI)	*P *	Adjusted OR (95 %CI)^a^	*P *	*P*_trend_^†^
rs2278008	A/A	145 (74.7)	109 (64.9)	Ref.		Ref.		**0.0407 **
	A/G	47 (24.2)	56 (33.3)	**0.63 (0.40–1.00)**	**0.0500 **	**0.51 (0.29–0.91)**	**0.0220 **	
	G/G	2 (1.0)	3 (1.8)	0.50 (0.08–3.05)	0.4535	0.42 (0.06–2.99)	0.3892	
	A/G + G/G	49 (25.3)	59 (35.1)	**0.62 (0.40–0.98)**	**0.0415 **	**0.51 (0.29–0.89)**	**0.0178 **	
							
	A allele	337 (86.9)	274 (81.5)	Ref.		Ref.		
	G allele	51 (13.1)	62 (18.5)	0.67 (0.45–1.00)	0.0506	**0.57 (0.35–0.93)**	**0.0251 **	

rs34677	G/G	143 (73.7)	123 (73.2)	Ref.		Ref.		0.8638
	G/T	46 (23.7)	43 (25.6)	0.92 (0.57–1.49)	0.7343	1.27 (0.66–2.42)	0.4761	
	T/T	5 (2.6)	2 (1.2)	2.15 (0.41–11.27)	0.3656	1.81 (0.25–12.93)	0.5559	
	G/T + T/T	51 (26.3)	45 (26.8)	0.97 (0.61–1.56)	0.9149	1.30 (0.70–2.44)	0.4069	
							
	G allele	332 (85.6)	289 (86.0)	Ref.		Ref.		
	T allele	56 (14.4)	47 (14.0)	1.04 (0.68–1.58)	0.8644	1.30 (0.74–2.27)	0.3656	

rs2287939	C/C	149 (76.8)	124 (73.8)	Ref.		Ref.		0.7474
	C/T	39 (20.1)	41 (24.4)	0.79 (0.48–1.30)	0.3587	0.66 (0.36–1.23)	0.1935	
	T/T	6 (3.1)	3 (1.8)	1.66 (0.41–6.79)	0.4777	0.78 (0.14–4.21)	0.7681	
	C/T + T/T	45 (23.2)	44 (26.2)	0.85 (0.53–1.37)	0.5095	0.67 (0.37–1.22)	0.1938	
							
	C allele	337 (86.9)	289 (86.0)	Ref.		Ref.		
	T allele	51 (13.1)	47 (14.0)	0.93 (0.61–1.43)	0.7403	0.72 (0.43–1.22)	0.2268	

rs10941112	G/G	69 (35.6)	76 (45.2)	Ref.		Ref.		0.2155
	G/A	96 (49.5)	66 (39.3)	**1.60 (1.02–2.52)**	**0.0410 **	1.52 (0.86–2.70)	0.1514	
	A/A	29 (14.9)	26 (15.5)	1.23 (0.66–2.29)	0.5164	1.24 (0.56–2.76)	0.5979	
	G/A + A/A	125 (64.4)	92 (54.8)	1.50 (0.98–2.28)	0.0616	1.44 (0.85–2.47)	0.1779	
							
	G allele	234 (60.3)	218 (64.9)	Ref.		Ref.		
	A allele	154 (39.7)	118 (35.1)	1.22 (0.90–1.65)	0.2055	1.21 (0.82–1.79)	0.3273	

rs3195676	G/G	69 (35.9)	74 (44.0)	Ref.		Ref.		0.2955
	G/A	94 (49.0)	68 (40.5)	1.48 (0.94–2.33)	0.0883	1.40 (0.79–2.48)	0.2553	
	A/A	29 (15.1)	26 (15.5)	1.20 (0.64–2.23)	0.5728	1.19 (0.53–2.66)	0.6729	
	G/A + A/A	123 (64.1)	94 (56.0)	1.40 (0.92–2.14)	0.1172	1.34 (0.78–2.29)	0.2849	
							
	G allele	232 (60.4)	216 (64.3)	Ref.		Ref.		
	A allele	152 (39.6)	120 (35.7)	1.18 (0.87–1.60)	0.2856	1.16 (0.79–1.72)	0.4404	

SNP: single nucleotide polymorphisms; OR: odds ratio; CI: confidence interval; AMACR: (R)-alpha-methyl-CoA racemase.

^a^Adjusted for age, smoking quantity, alcohol drinking, and family history of prostate cancer.

^†^Cochran-Armitage trend test for the number of variant alleles.

**Table 3:** Association between AMACR (5p13) polymorphisms and clinical factors in prostate cancer patients.

rs2278008	AG + GG (%)	AA (%)		OR (95% CI)	*P *	Adjusted OR (95% CI)^a^	*P *	G (%)	A (%)	OR (95% CI)	*P *	Adjusted OR (95% CI)^a^	*P *
Gleason score													
<7	29 (59.2)	70 (48.3)		Ref.		Ref.		30 (58.8)	168 (49.9)	Ref.		Ref.	
≥7	20 (40.8)	75 (51.7)		0.64 (0.33–1.24)	0.1883	0.63 (0.32–1.24)	0.1845	21 (41.2)	169 (50.1)	0.70 (0.38–1.26)	0.2340	0.69 (0.38–1.28)	0.2418
pTstage													
≤pT2b	12 (24.5)	34 (23.4)		Ref.		Ref.		12 (23.5)	80 (23.7)	Ref.		Ref.	
≥pT2c	37 (75.5)	111 (76.6)		0.94 (0.44–2.01)	0.8822	1.00 (0.47–2.15)	0.9904	39 (76.5)	257 (76.3)	1.01 (0.51–2.02)	0.9739	1.07 (0.53–2.15)	0.8487

rs34677	GT + TT (%)	GG (%)						T (%)	G (%)				

Gleason score													
<7	26 (51.0)	73 (51.0)		Ref.		Ref.		30 (53.6)	168 (50.6)	Ref.		Ref.	
≥7	25 (49.0)	70 (49.0)		1.00 (0.53–1.90)	0.9933	1.06 (0.55–2.05)	0.8558	26 (46.4)	164 (49.4)	0.89 (0.50–1.57)	0.6811	0.93 (0.52–1.66)	0.7990
pTstage													
≤pT2b	18 (35.3)	28 (19.6)		Ref.		Ref.		20 (35.7)	72 (21.7)	Ref.		Ref.	
≥pT2c	33 (64.7)	115 (80.4)		0.45 (0.22–0.91)	**0.0254 **	0.42 (0.21–0.87)	**0.0193 **	36 (64.3)	260 (78.3)	0.50 (0.27–0.91)	**0.0243 **	0.48 (0.26–0.89)	**0.0191 **

rs2287939	CT + TT (%)	CC (%)						T (%)	C (%)				

Gleason score													
<7	27 (60.0)	72 (48.3)		Ref.		Ref.		31 (60.8)	167 (49.6)	Ref.		Ref.	
≥7	18 (40.0)	77 (51.7)		0.62 (0.32–1.23)	0.1716	0.52 (0.25–1.05)	0.0686	20 (39.2)	170 (50.4)	0.63 (0.35–1.16)	0.1372	0.54 (0.29–1.01)	0.0536
pTstage													
≤pT2b	12 (26.7)	34 (22.8)		Ref.		Ref.		14 (27.5)	78 (23.1)	Ref.		Ref.	
≥pT2c	33 (73.3)	115 (77.2)		0.81 (0.38–1.74)	0.5952	0.84 (0.38–1.81)	0.6492	37 (72.5)	259 (76.9)	0.80 (0.41–1.55)	0.5011	0.83 (0.42–1.64)	0.5966

rs10941112	GA + AA (%)	GG (%)						A (%)	G (%)				

Gleason score													
<7	54 (43.2)	45 (65.2)		Ref.		Ref.		70 (45.5)	128 (54.7)	Ref.		Ref.	
≥7	71 (56.8)	24 (34.8)		2.47 (1.34–4.53)	**0.0037 **	2.28 (1.22–4.29)	**0.0101 **	84 (54.5)	106 (45.3)	1.45 (0.96–2.18)	0.0751	1.39 (0.91–2.11)	0.1272
pTstage													
≤pT2b	22 (17.6)	24 (34.8)		Ref.		Ref.		28 (18.2)	64 (27.4)	Ref.		Ref.	
≥pT2c	103 (82.4)	45 (65.2)		2.50 (1.27–4.91)	**0.0080 **	2.60 (1.30–5.21)	**0.0071 **	126 (81.8)	170 (72.6)	1.69 (1.03–2.79)	**0.0389 **	1.77 (1.07–2.95)	**0.0272 **

rs3195676	GA + AA (%)	GG (%)						A (%)	G (%)				

Gleason score													
<7	54 (43.9)	45 (65.2)		Ref.		Ref.		70 (46.1)	128 (55.2)	Ref.		Ref.	
≥7	69 (56.1)	24 (34.8)		2.40 (1.30–4.41)	**0.0050 **	2.35 (1.26–4.41)	**0.0076 **	82 (53.9)	104 (44.8)	1.44 (0.96–2.17)	0.0808	1.39 (0.92–2.12)	0.1203
pTstage													
≤pT2b	22 (17.9)	24 (34.8)		Ref.		Ref.		28 (18.4)	64 (27.6)	Ref.		Ref.	
≥pT2c	101 (82.1)	45 (65.2)		2.45 (1.24–4.82)	**0.0095 **	2.66 (1.33–5.34)	**0.0057 **	124 (81.6)	168 (72.4)	1.69 (1.02–2.78)	**0.0408 **	1.78 (1.07–2.95)	**0.0259 **

pTstage: pathologic stage; OR: odds ratio; 95% CI: 95% confidence interval; AMACR: (R)-alpha-methyl-CoA racemase.

^a^Adjusted for age, smoking quantity, alcohol drinking, and family history of prostate cancer.

**Table 4:** Association analysis of AMACR (5p13) polymorphisms with AMACR expressing prostate cancer risk.

SNP	Genotype	Cases (%)	Controls (%)	Crude OR (95%CI)	*P *	Adjusted OR (95%CI)^a^	*P *	P_trend_^†^
rs2278008	A/A	120 (77.9)	109 (64.9)	Ref.		Ref.		0.0088
	A/G	33 (21.4)	56 (33.3)	0.54 (0.32–0.88)	0.0147	0.48 (0.26–0.89)	0.0207	
	G/G	1 (0.6)	3 (1.8)	0.30 (0.03–2.95)	0.3040	0.37 (0.04–3.92)	0.4091	
	A/G + G/G	34 (22.1)	59 (35.1)	0.52 (0.32–0.86)	0.0104	0.47 (0.26–0.87)	0.0167	
							
	A allele	246 (87.9)	274 (81.5)	Ref.		Ref.		
	G allele	34 (12.1)	62 (18.5)	0.61 (0.39 –0.96)	0.0327	0.59 (0.34–1.02)	0.0598	

rs34677	G/G	113 (73.4)	123 (73.2)	Ref.		Ref.		0.7336
	G/T	36 (23.4)	43 (25.6)	0.91 (0.55 –1.52)	0.7217	1.33 (0.66–2.67)	0.4217	
	T/T	5 (3.2)	2 (1.2)	2.72 (0.52–14.29)	0.2373	2.28 (0.29–8.15)	0.4354	
	G/T + T/T	41 (26.6)	45 (26.8)	0.99 (0.60–1.63)	0.9738	1.40 (0.71–2.73)	0.3300	
							
	G allele	239 (85.4)	289 (86.0)	Ref.		Ref.		
	T allele	41 (14.6)	47 (14.0)	1.05 (0.67–1.66)	0.8169	1.35 (0.72–2.50)	0.3479	

rs2287939	C/C	122 (79.2)	124 (73.8)	Ref.		Ref.		0.2589
	C/T	30 (19.5)	41 (24.4)	0.74 (0.44–1.27)	0.2763	0.72 (0.37–1.38)	0.3207	
	T/T	2 (1.3)	3 (1.8)	0.68 (0.11–4.13)	0.6728	0.50 (0.05–4.88)	0.5550	
	C/T + T/T	32 (20.8)	44 (26.2)	0.74 (0.44–1.24)	0.2542	0.70 (0.37–1.33)	0.2775	
							
	C allele	247 (88.2)	289 (86.0)	Ref.		Ref.		
	T allele	33 (11.8)	47 (14.0)	0.82 (0.51–1.32)	0.4186	0.79 (0.44–1.42)	0.4274	

rs10941112	G/G	58 (37.7)	76 (45.2)	Ref.		Ref.		0.4154
	G/A	74 (48.1)	66 (39.3)	1.47 (0.91–2.37)	0.1134	1.45 (0.79–2.66)	0.2322	
	A/A	22 (14.3)	26 (15.5)	1.11 (0.57–2.15)	0.7601	1.08 (0.45–2.57)	0.8655	
	G/A + A/A	96 (62.3)	92 (54.8)	1.37 (0.88–2.13)	0.1688	1.35 (0.76–2.38)	0.3041	
							
	G allele	107 (38.2)	118 (35.1)	Ref.		Ref.		
	A allele	173 (61.8)	218 (64.9)	1.14 (0.82–1.59)	0.4271	1.12 (0.73–1.71)	0.6157	

rs3195676	G/G	58 (37.9)	74 (44.0)	Ref.		Ref.		0.5202
	G/A	73 (47.7)	68 (40.5)	1.37 (0.85–2.21)	0.1959	1.34 (0.73–2.46)	0.3503	
	A/A	22 (14.4)	26 (15.5)	1.08 (0.56–2.10)	0.8211	1.03 (0.43–2.46)	0.9503	
	G/A + A/A	95 (62.1)	94 (56.0)	1.29 (0.82–2.02)	0.2646	1.25 (0.71–2.22)	0.4360	
							
	G allele	106 (38.1)	120 (35.7)	Ref.		Ref.		
	A allele	172 (61.9)	216 (64.3)	1.11 (0.80–1.54)	0.5364	1.07 (0.70–1.64)	0.7577	

SNP: single nucleotide polymorphisms; OR: odds ratio; CI: confidence interval.

^a^Adjusted for age, smoking quantity, alcohol drinking, and family history of prostate cancer.

^†^Cochran-Armitage trend test for the number of variant alleles.

**Table 5:** Association between AMACR (5p13) polymorphisms and prognostic factors in prostate cancer patients in AMACR expressing subgroup.

rs2278008	AG + GG (%)	AA (%)	OR (95% CI)	*P *	Adjusted OR (95% CI)^a^	*P *	G (%)		A (%)	OR (95% CI)			*P *	Adjusted OR (95% CI)^a^	*P *
Gleason score															
<7	23 (67.6)	59 (49.2)					22 (64.7)		130 (52.8)						
≥7	11 (32.4)	61 (50.8)	0.46 (0.21–1.03)	0.0598	0.43 (0.19–0.99)	0.0482	12 (35.3)		116 (47.2)	0.61 (0.29–1.29)			0.1963	0.59 (0.28–1.28)	0.1823
pTstage															
≤pT2b	7 (20.6)	28 (23.3)					7 (20.6)		53 (21.5)						
≥pT2c	27 (79.4)	92 (76.7)	1.17 (0.46–2.98)	0.7362	1.24 (0.48–3.18)	0.6530	27 (79.4)		193 (78.5)	1.06 (0.44–2.57)			0.8986	1.12 (0.46–2.74)	0.7986

rs34677	GT + TT (%)	GG (%)					T (%)		G (%)						

Gleason score															
<7	21 (51.2)	61 (54.0)					21 (51.2)		131 (54.8)						
≥7	20 (48.8)	52 (46.0)	1.12 (0.55–2.28)	0.7614	1.20 (0.57–2.53)	0.6249	20 (48.8)		108 (45.2)	1.16 (0.60–2.24)			0.6698	1.22 (0.61–2.42)	0.5751
pTstage															
≤pT2b	15 (36.6)	20 (17.7)					14 (34.1)		46 (19.2)						
≥pT2c	26 (63.4)	93 (82.3)	0.37 (0.17–0.83)	0.0154	0.35 (0.15–0.79)	0.0119	27 (65.9)		193 (80.8)	0.46 (0.22–0.95)			0.0346	0.43 (0.21–0.91)	0.0281

rs2287939	CT + TT (%)	CC (%)					T (%)		C (%)						

Gleason score															
<7	19 (59.4)	63 (51.6)					19 (57.6)		133 (53.8)						
≥7	13 (40.6)	59 (48.4)	0.73 (0.33–1.61)	0.4360	0.62 (0.27–1.44)	0.2697	14 (42.4)		114 (46.2)	0.86 (0.41–1.79)			0.6865	0.76 (0.35–1.64)	0.4819
pTstage															
≤pT2b	7 (21.9)	28 (23.0)					7 (21.2)		53 (21.5)						
≥pT2c	25 (78.1)	94 (77.0)	1.06 (0.42–2.72)	0.8972	1.07 (0.41–2.77)	0.8944	26 (78.8)		194 (78.5)	1.01 (0.42–2.47)			0.9743	1.02 (0.41–2.50)	0.9722

rs10941112	GA + AA (%)	GG (%)					A (%)		G (%)						

Gleason score															
<7	46 (47.9)	36 (62.1)					55 (51.4)		97 (56.1)						
≥7	50 (52.1)	22 (37.9)	1.78 (0.92–3.46)	0.0895	1.73 (0.87–3.45)	0.1165	52 (48.6)		76 (43.9)	1.21 (0.74–1.96)			0.4464	1.18 (0.72–1.93)	0.5217
pTstage															
≤pT2b	15 (15.6)	20 (34.5)					15 (14.0)		45 (26.0)						
≥pT2c	81 (84.4)	38 (65.5)	2.84 (1.31–6.15)	0.0080	2.97 (1.35–6.52)	0.0066	92 (86.0)		128 (74.0)	2.16 (1.13–4.10)			0.0191	2.23 (1.17–4.27)	0.0153

rs3195676	GA + AA (%)	GG (%)					A (%)		G (%)						

Gleason score															
<7	46 (48.4)	36 (62.1)					55 (51.9)		97 (56.4)						
≥7	49 (51.6)	22 (37.9)	1.74 (0.90–3.39)	0.1019	1.71 (0.86–3.40)	0.1289	51 (48.1)		75 (43.6)	1.20 (0.74–1.95)			0.4635	1.17 (0.71–1.93)	0.5294
pTstage															
≤pT2b	15 (15.8)	20 (34.5)					15 (14.2)		45 (26.2)						
≥pT2c	80 (84.2)	38 (65.5)	2.81 (1.30–6.08)	0.0089	2.94 (1.34–6.45)	0.0071	91 (85.8)		127 (73.8)	2.15 (1.13–4.09)			0.0197	2.23 (1.16–4.27)	0.0155

pTstage: pathologic stage; OR: odds ratio; 95% CI: 95% confidence interval.

^a^Adjusted for age, smoking quantity, alcohol drinking, and family history of prostate cancer.

**Table 6:** AMACR (5p13) haplotypes and their association with prostate cancer risk.

Haplotype*	Cases (%)	Controls (%)	Crude OR (95% CI)	*P* value	Adjusted OR(95% CI)^a^	*P* value
12345-1[AGCAA]	147 (37.9)	116 (34.3)	Ref.		Ref.	
12345-2[AGCGG]	116 (29.9)	103 (30.5)	0.89 (0.62–1.27)	0.5208	0.95 (0.60–1.50)	0.8205
12345-3[ATCGG]	56 (14.4)	47 (13.9)	0.94 (0.60–1.49)	0.7919	1.14 (0.62–2.10)	0.6743
12345-4[GGTGG]	33 (8.5)	39 (11.5)	0.67 (0.40–1.13)	0.1306	0.59 (0.31–1.13)	0.1108
**12345-5[GGCGG]**	11 (2.8)	20 (5.9)	**0.43 (0.20–0.94)**	**0.0348**	**0.41 (0.17–1.00)**	**0.0489**
12345-6[AGTGG]	18 (4.6)	9 (2.7)	1.58 (0.68–3.64)	0.2849	0.97 (0.35–2.74)	0.9603
12345-7[GGCAA]	7 (1.8)	3 (0.9)	1.84 (0.47–7.28)	0.3839	1.54 (0.28–8.36)	0.6190
**Haplotype pair**						
** 12-2 [GG]**						
0 copies	145 (74.7)	110 (65.1)	Ref.		Ref.	
1 or 2 copies	49 (25.3)	59 (34.9)	**0.63 (0.40–0.99)**	**0.0455 **	**0.51 (0.29–0.90)**	**0.0199 **
** 14-3 [GG]**						
0 copies	153 (78.9)	112 (66.3)	Ref.		Ref.	
1 or 2 copies	41 (21.1)	57 (33.7)	**0.53 (0.33 –0.84)**	**0.0074 **	**0.43 (0.24–0.77)**	**0.0047 **
** 15-3 [GG]**						
0 copies	150 (77.3)	112 (66.3)	Ref.		Ref.	
1 or 2 copies	44 (22.7)	57 (33.7)	**0.58 (0.36–0.92)**	**0.0197 **	**0.47 (0.27 –0.84)**	**0.0104 **
** 145-3 [GGG]**						
0 copies	150 (77.3)	112 (66.3)	Ref.		Ref.	
1 or 2 copies	44 (22.7)	57 (33.7)	**0.58 (0.36–0.92)**	**0.0197 **	**0.47 (0.27 –0.84)**	**0.0104 **
** 1245-4 [GGGG]**						
0 copies	151 (77.8)	113 (66.9)	Ref.		Ref.	
1 or 2 copies	43 (22.2)	56 (33.1)	**0.57 (0.36–0.92)**	**0.0199 **	**0.48 (0.27 –0.86)**	**0.0132 **
** 1345-4 [GCGG]**						
0 copies	182 (93.8)	148 (87.6)	Ref.		Ref.	
1 or 2 copies	12 (6.2)	21 (12.4)	**0.46 (0.22–0.98)**	**0.0428 **	**0.40 (0.17–0.95)**	**0.0379 **
** 12345-5 [GGCGG]**						
0 copies	183 (94.3)	149 (88.2)	Ref.		Ref.	
1 or 2 copies	11 (5.7)	20 (11.8)	**0.45 (0.21–0.96)**	**0.0400 **	**0.41 (0.17–0.99)**	**0.0472 **

Haplotypes with total frequencies of less than 1 percent were excluded in table.

OR: odds ratio; CI: confidence interval; AMACR: (R)-alpha-methyl-CoA racemase.

*1: rs2278008; 2: rs34677; 3: rs2287939; 4: rs10941112; 5: rs3195676.

^a^Adjusted for age, smoking quantity, alcohol drinking, and family history of prostate cancer.

## Abbreviations

AMACR: (R)-Alpha-methyl-CoA racemase
SNP: Single nucleotide polymorphism
GWAS: Genome-wide association study
HWE: Hardy-Weinberg Equilibrium
CIs: Confidence intervals
LD: Linkage disequilibrium.
