# Supplementary material for: Genetic Variations of α-Methylacyl-CoA Racemase Are Associated with Sporadic Prostate Cancer Risk in Ethnically Homogenous Koreans
Source: Biomed Res Int. 2013 Dec 7;2013:394285. doi: 10.1155/2013/394285 (PMC3870614; doi:10.1155/2013/394285)
Supplement: Supplementary file 1 — We collected the information of all coding SNPs in AMACR with heterozygosity > 0; a total of 17 SNPs were identified (Supplemental Table 1). Primers for 11 SNPs were designed using DESINGER (Sequenom, CA, USA) software (Supplemental Table 2). Genotype frequencies for all of the target SNPs of AMACR in this study are presented in Supplemental Table 3. Supplemental Table 4 shows the relation between variants of AMACR and intensity of AMACR expression. Supplemental Table 5 represents the AMACR (5p13) haplotypes and their association with prostate cancer risk. Supplemental figure describes results of immunohistochemical staining of AMACR according to variant genotypes of rs2278008. [file 394285.f1.docx]

**Supplemental Table1. SNPs investigated in this study.**

| **rs number** | **SNP position** | **Alleles** | **Amino acid change** | **Amino acid position** | **Remarks** |
| --- | --- | --- | --- | --- | --- |
| rs1055202 | 33989367 | C/T | P/L | 327 |  |
| rs113361629 | 33989403 | T/A | F/Y | 315 | n.i * |
| rs35655488 | 33989510 | G/A | T/T | 279 |  |
| **rs2278008** | 33989518 | G/A | E/K | 277 |  |
| rs3195678 | 33989565 | T/C | M/T | 261 |  |
| rs113452265 | 33989586 | A/G | E/G | 254 | n.i * |
| rs113345038 | 33998764 | T/C | Y/H | 241 | n.i * |
| **rs34677** | 33998768 | G/T | Q/H | 239 |  |
| rs9282594 | 33998773 | C/T | P/S | 238 |  |
| rs76184600 | 33998787 | T/G | V/G | 233 | Design Fail |
| rs11540908 | 33998810 | A/G | T/T | 225 |  |
| **rs2287939** | 33998883 | T/C | L/S | 201 |  |
| **rs10941112** | 34004707 | G/A | G/D | 175 |  |
| rs117220551 | 34004723 | G/T | D/Y | 170 | Design Fail |
| rs113840552 | 34004797 | C/T | V/A | 145 | n.i * |
| rs16892150 | 34005899 | G/A | R/Q | 118 |  |
| **rs3195676** | 34008100 | G/A | V/M | 9 |  |

SNP, single nucleotide polymorphism

* Not included because of heterozygosity 0.5

Design Fail, unable to read sequences with designed primers

**Supplemental Table 2. Target SNPs of AMACR for experiment and sequences of the PCR primers.**

| **SNP_ID** | **Forward PCR Primer** | **Reverse PCR Primer** | **AMP_LEN** | **Extension Primer** |
| --- | --- | --- | --- | --- |
| rs1055202 | ACGTTGGATGTTATCACCAGTGAGGAGCAG | ACGTTGGATGTTGAAAGAAGGGATGGCTGG | 99 | TTAACAGCAGAGGTGCA |
| rs35655488 | ACGTTGGATGGCCGTCAAAGATTTGACACC | ACGTTGGATGGATGAGCATGGATGATTGGC | 114 | AGATGTATTTGCAGAGAAGAC |
| rs2278008 | ACGTTGGATGGATGAGCATGGATGATTGGC | ACGTTGGATGTTTGACACCACTCTGCCTTC | 103 | CTCTGCCTTCGTCTTCT |
| rs3195678 | ACGTTGGATGGTCTGATGAACTTCCCAATC | ACGTTGGATGTCTGCCTTCGTCTTCTCTGC | 113 | TTTCTGGCCAATCATCC |
| rs34677 | ACGTTGGATGACGCGTGAAGTTCACTTACC | ACGTTGGATGGGAATTCATGGCTGTTGGAG | 95 | GGAGCAATAGAACCCCA |
| rs9282594 | ACGTTGGATGTACGACTTACAGGACAGCAG | ACGTTGGATGCTTTGATCAGCAGCTCGTAG | 100 | GCAGCTCGTAGAACTGGG |
| rs11540908 | ACGTTGGATGGGTGGAGCACCTTTCTATAC | ACGTTGGATGGGGTTCTATTGCTCCAACAG | 92 | GCCATGAATTCCCCATCTGC |
| rs2287939 | ACGTTGGATGTCCAACATGTTCTGTCCTCG | ACGTTGGATGTCAAAGGTGGAAGGAACAGC | 115 | CTGTGGAAAACTCAGAAAT |
| rs10941112 | ACGTTGGATGATGGCTCTTTTTGACCGCAC | ACGTTGGATGACAAGTGGCAGGCATCTACC | 112 | TCAATGACCTGACCCTTG |
| rs16892150 | ACGTTGGATGGAGTGGATTTGGCCAGTCAG | ACGTTGGATGTCAACATACCTGACAAAGCC | 100 | GATATCGTGGCCAGCTAAC |
| rs3195676 | ACGTTGGATGGGACCATAGCACAGAACGG | ACGTTGGATGCATGGCACTGCAGGGCATC | 91 | ACTGCAGGGCATCTCGGTC |

SNP, single nucleotide polymorphism; AMACR, (R)-alpha-methyl-CoA racemase

**Supplemental Table 3. Frequency of the target SNPs of AMACR for the experiment.**

| **rs number** | **Case** | | | | | |  | **Control** | | | | | | **Remarks** |
| --- | --- | --- | --- | --- | --- | --- | --- | --- | --- | --- | --- | --- | --- | --- |
|  | **Genotype** | **Freq.** | **Genotype** | **Freq.** | **Genotype** | **Freq.** |  | **Genotype** | **Freq.** | **Genotype** | **Freq.** | **Genotype** | **Freq.** |  |
| rs1055202 | CC | 194 | CT | 0 | TT | 0 |  | CC | 170 | CT | 0 | TT | 0 | mono.* |
| rs35655488 | GG | 194 | GA | 0 | AA | 0 |  | GG | 169 | GA | 0 | AA | 0 | mono.* |
| rs2278008 | GG | 2 | GA | 47 | AA | 145 |  | GG | 3 | GA | 56 | AA | 109 |  |
| rs34677 | GG | 143 | GT | 46 | TT | 5 |  | GG | 123 | GT | 43 | TT | 2 |  |
| rs9282594 | CC | 194 | CT | 0 | TT | 0 |  | CC | 169 | CT | 0 | TT | 0 | mono.* |
| rs11540908 | AA | 194 | AG | 0 | GG | 0 |  | AA | 169 | AG | 0 | GG | 0 | mono.* |
| rs2287939 | TT | 6 | TC | 39 | CC | 149 |  | TT | 3 | TC | 41 | CC | 124 |  |
| rs10941112 | GG | 69 | GA | 96 | AA | 29 |  | GG | 76 | GA | 66 | AA | 26 |  |
| rs16892150 | GG | 193 | GA | 0 | AA | 0 |  | GG | 169 | GA | 0 | AA | 0 | mono.* |
| rs3195676 | GG | 69 | GA | 94 | AA | 29 |  | GG | 74 | GA | 68 | AA | 26 |  |

SNP, single nucleotide polymorphism; AMACR, (R)-alpha-methyl-CoA racemase

***SNPs revealed as mono**morphic in Korean subjects

**Supplemental Table 4. Relation between variants of AMACR and intensity of AMACR expression**

| **SNP** | **Genotype** | **Immunohistochemistry of AMACR** | | | | | | | | **P-value*** |
| --- | --- | --- | --- | --- | --- | --- | --- | --- | --- | --- |
|  |  | **No**  **staining (%)** | | **Weakly**  **positive (%)** | | **Positive (%)** | | **Strongly**  **positive (%)** | |  |
| rs2278008 | A/A | 24 | (61.5) | 25 | (73.5) | 44 | (80.0) | 51 | (78.5) | 0.3871 |
|  | G/A | 14 | (35.9) | 9 | (26.5) | 11 | (20.0) | 13 | (20.0) |  |
|  | G/G | 1 | (2.6) | 0 | (0.0) | 0 | (0.0) | 1 | (1.5) |  |
| rs34677 | G/G | 29 | (74.4) | 25 | (73.5) | 44 | (80.0) | 44 | (67.7) | 0.3190 |
|  | G/T | 10 | (25.6) | 8 | (23.5) | 8 | (14.6) | 20 | (30.8) |  |
|  | T/T | 0 | (0.0) | 1 | (2.9) | 3 | (5.5) | 1 | (1.5) |  |
| rs2287939 | C/C | 27 | (69.2) | 23 | (67.7) | 46 | (83.6) | 53 | (81.5) | 0.0396 |
|  | C/T | 8 | (20.5) | 11 | (32.4) | 8 | (14.6) | 11 | (16.9) |  |
|  | T/T | 4 | (10.3) | 0 | (0.0) | 1 | (1.8) | 1 | (1.5) |  |
| rs10941112 | A/A | 7 | (18.0) | 5 | (14.7) | 8 | (14.6) | 9 | (13.9) | 0.9666 |
|  | G/A | 21 | (53.9) | 17 | (50.0) | 26 | (47.3) | 31 | (47.7) |  |
|  | G/G | 11 | (28.2) | 12 | (35.3) | 21 | (38.2) | 25 | (38.5) |  |
| rs3195676 | A/A | 7 | (18.4) | 5 | (15.2) | 8 | (14.6) | 9 | (13.9) | 0.9761 |
|  | A/G | 20 | (52.6) | 16 | (48.5) | 26 | (47.3) | 31 | (47.7) |  |
|  | G/G | 11 | (29.0) | 12 | (36.4) | 21 | (38.2) | 25 | (38.5) |  |

*using Pearson’s χ^2^ test

**Supplemental Table 5.** AMACR (5p13) haplotypes and their association with prostate cancer risk**.**

| **Haplotype*** | | **Cases** | **(%)** | | **Controls** | **(%)** | **Crude OR** | **( 95%CI)** | | ***P*-value** |  | **Adjusted**  **OR** | **( 95%CI) ^a^** | | | ***P*-value ^a^** | |  |
| --- | --- | --- | --- | --- | --- | --- | --- | --- | --- | --- | --- | --- | --- | --- | --- | --- | --- | --- |
| 12-1[AG] | | 281 | (72.4) | | 229 | (67.8) | Ref. |  |  |  |  | Ref. |  | |  |  | |  |
| **12-2[GG]** | | 51 | (13.1) | | 62 | (18.3) | **0.67** | **(0.45 -** | **1.01)** | **0.0556** |  | **0.59** | **(0.36 -** | | **0.97)** | **0.0387** | |  |
| 12-3[AT] | | 56 | (14.4) | | 47 | (13.9) | 0.97 | (0.64 - | 1.49) | 0.8921 |  | 1.18 | (0.67 - | | 2.09) | 0.5742 | |  |
|  | |  |  | |  |  |  |  |  |  |  |  |  | |  |  | |  |
| 13-1[AC] | | 319 | (82.2) | | 267 | (79.0) | Ref. |  |  |  |  | Ref. |  | |  |  | |  |
| 13-2[GT] | | 33 | (8.5) | | 39 | (11.5) | 0.71 | (0.43 - | 1.16) | 0.1687 |  | 0.60 | (0.33 - | | 1.08) | 0.0899 | |  |
| 13-3[GC] | | 18 | (4.6) | | 23 | (6.8) | 0.66 | (0.35 - | 1.24) | 0.1936 |  | 0.55 | (0.26 - | | 1.16) | 0.1177 | |  |
| 13-4[AT] | | 18 | (4.6) | | 9 | (2.7) | 1.67 | (0.74 - | 3.79) | 0.2163 |  | 0.98 | (0.36 - | | 2.68) | 0.9649 | |  |
|  | |  |  | |  |  |  |  |  |  |  |  |  | |  |  | |  |
| 14-1[AG] | | 180 | (48.9) | | 160 | (47.3) | Ref. |  |  |  |  | Ref. |  | |  |  | |  |
| 14-2[AA] | | 141 | (38.3) | | 116 | (34.3) | 1.08 | (0.78 - | 1.50) | 0.6409 |  | 1.03 | (0.68 - | | 1.57) | 0.8875 | |  |
| **14-3[GG]** | | 42 | (11.4) | | 60 | (17.8) | **0.62** | **(0.40 -** | **0.97)** | **0.0380** |  | **0.53** | **(0.31 -** | | **0.91)** | **0.0210** | |  |
| 14-4[GA] | | 5 | (1.4) | | 2 | (0.6) | 2.22 | (0.43 - | 11.60) | 0.3443 |  | 2.11 | (0.22 - | | 20.32) | 0.5169 | |  |
|  | |  |  | |  |  |  |  |  |  |  |  |  | |  |  | |  |
| 15-1[AG] | | 191 | (49.2) | | 158 | (46.8) | Ref. |  |  |  |  | Ref. |  | |  |  | |  |
| 15-2[AA] | | 146 | (37.6) | | 118 | (34.9) | 1.02 | (0.74 - | 1.41) | 0.8873 |  | 0.97 | (0.64 - | | 1.47) | 0.8762 | |  |
| **15-3[GG]** | | 45 | (11.6) | | 60 | (17.8) | **0.62** | **(0.40 -** | **0.96)** | **0.0336** |  | **0.52** | **(0.30 -** | | **0.89)** | **0.0160** | |  |
| 15-4[GA] | | 6 | (1.6) | | 2 | (0.6) | 2.48 | (0.49 - | 12.47) | 0.2697 |  | 2.65 | (0.29 - | | 24.04) | 0.3857 | |  |
|  | |  |  | |  |  |  |  |  |  |  |  |  | |  |  | |  |
| 123-1[AGC] | | 263 | (67.8) | | 220 | (65.1) | Ref. |  |  |  |  | Ref. |  | |  |  | |  |
| 123-2[ATC] | | 56 | (14.4) | | 47 | (13.9) | 1.00 | (0.65 - | 1.53) | 0.9878 |  | 1.18 | (0.66 - | | 2.09) | 0.5759 | |  |
| 123-3[GGT] | | 33 | (8.5) | | 39 | (11.5) | 0.71 | (0.43 - | 1.16) | 0.1729 |  | 0.61 | (0.33 - | | 1.12) | 0.1109 | |  |
| 123-4[GGC] | | 18 | (4.6) | | 23 | (6.8) | 0.66 | (0.34 - | 1.24) | 0.1961 |  | 0.57 | (0.27 - | | 1.20) | 0.1386 | |  |
| 123-5[AGT] | | 18 | (4.6) | | 9 | (2.7) | 1.67 | (0.74 - | 3.80) | 0.2187 |  | 1.00 | (0.36 - | | 2.77) | 0.9936 | |  |
|  | |  |  | |  |  |  |  |  |  |  |  |  | |  |  | |  |
| 124-1[AGA] | | 147 | (37.9) | | 116 | (34.3) | Ref. |  |  |  |  | Ref. |  | |  |  | |  |
| 124-2[AGG] | | 134 | (34.5) | | 113 | (33.4) | 0.94 | (0.66 - | 1.33) | 0.7094 |  | 0.94 | (0.60 - | | 1.47) | 0.7826 | |  |
| 124-3[ATG] | | 56 | (14.4) | | 47 | (13.9) | 0.94 | (0.60 - | 1.49) | 0.7919 |  | 1.14 | (0.62 - | | 2.11) | 0.6709 | |  |
| **124-4[GGG]** | | 44 | (11.3) | | 59 | (17.5) | **0.59** | **(0.37 -** | **0.93)** | **0.0239** |  | **0.53** | **(0.30 -** | | **0.92)** | **0.0250** | |  |
| 124-5[GGA] | | 7 | (1.8) | | 3 | (0.9) | 1.84 | (0.47 - | 7.27) | 0.3842 |  | 1.54 | (0.28 - | | 8.37) | 0.6185 | |  |
|  | |  |  | |  |  |  |  |  |  |  |  |  | |  |  | |  |
| 125-1[AGA] | | 145 | (37.4) | | 117 | (34.6) | Ref. |  |  |  |  | Ref. |  | |  |  | |  |
| 125-2[AGG] | | 136 | (35.1) | | 112 | (33.1) | 0.98 | (0.69 - | 1.39) | 0.9088 |  | 0.97 | (0.62 - | | 1.51) | 0.8843 | |  |
| 125-3[ATG] | | 56 | (14.4) | | 47 | (13.9) | 0.96 | (0.61 - | 1.52) | 0.8662 |  | 1.16 | (0.63 - | | 2.14) | 0.6363 | |  |
| **125-4[GGG]** | | 44 | (11.3) | | 59 | (17.5) | **0.60** | **(0.38 -** | **0.95)** | **0.0305** |  | **0.54** | **(0.31 -** | | **0.94)** | **0.0284** | |  |
| 125-5[GGA] | | 7 | (1.8) | | 3 | (0.9) | 1.88 | (0.48 - | 7.44) | 0.3671 |  | 1.56 | (0.29 - | | 8.49) | 0.6065 | |  |
|  | |  |  | |  |  |  |  |  |  |  |  |  | |  |  | |  |
| 134-1[ACG] | | 171 | (44.1) | | 151 | (44.7) | Ref. |  |  |  |  | Ref. |  | |  |  | |  |
| 134-2[ACA] | | 148 | (38.1) | | 116 | (34.3) | 1.13 | (0.81 - | 1.56) | 0.4749 |  | 1.02 | (0.67 - | | 1.55) | 0.9413 | |  |
| 134-3[GTG] | | 33 | (8.5) | | 39 | (11.5) | 0.75 | (0.45 - | 1.25) | 0.2652 |  | 0.60 | (0.32 - | | 1.12) | 0.1106 | |  |
| **134-4[GCG]** | | 12 | (3.1) | | 21 | (6.2) | **0.51** | **(0.24 -** | **1.06)** | **0.0709** |  | **0.41** | **(0.17 -** | | **0.97)** | **0.0413** | |  |
| 134-5[ATG] | | 18 | (4.6) | | 9 | (2.7) | 1.77 | (0.77 - | 4.05) | 0.1790 |  | 0.99 | (0.35 - | | 2.75) | 0.9775 | |  |
| 134-6[GCA] | | 6 | (1.6) | | 2 | (0.6) | 2.65 | (0.53 - | 13.32) | 0.2371 |  | 2.71 | (0.30 - | | 24.55) | 0.3766 | |  |
|  | |  |  | |  |  |  |  |  |  |  |  |  | |  |  | |  |
| 135-1[ACG] | | 173 | (44.6) | | 149 | (44.1) | Ref. |  |  |  |  | Ref. |  | |  |  | |  |
| 135-2[ACA] | | 146 | (37.6) | | 118 | (34.9) | 1.07 | (0.77 - | 1.48) | 0.7031 |  | 0.96 | (0.63 - | | 1.47) | 0.8670 | |  |
| 135-3[GTG] | | 33 | (8.5) | | 39 | (11.5) | 0.73 | (0.44 - | 1.22) | 0.2265 |  | 0.59 | (0.31 - | | 1.10) | 0.0956 | |  |
| **135-4[GCG]** | | 12 | (3.1) | | 21 | (6.2) | **0.49** | **(0.23 -** | **1.03)** | **0.0612** |  | **0.40** | **(0.17 -** | | **0.94)** | **0.0364** | |  |
| 135-5[ATG] | | 18 | (4.6) | | 9 | (2.7) | 1.72 | (0.75 - | 3.95) | 0.1989 |  | 0.96 | (0.35 - | | 2.69) | 0.9420 | |  |
| 135-6[GCA] | | 6 | (1.6) | | 2 | (0.6) | 2.58 | (0.51 - | 12.99) | 0.2494 |  | 2.64 | (0.29 - | | 23.98) | 0.3880 | |  |
|  | |  |  | |  |  |  |  |  |  |  |  |  | |  |  | |  |
| 145-1[AGG] | | 189 | (48.7) | | 158 | (46.8) | Ref. |  |  |  |  | Ref. |  | |  |  | |  |
| 145-2[AAA] | | 148 | (38.1) | | 117 | (34.6) | 1.06 | (0.77 - | 1.46) | 0.7334 |  | 0.98 | (0.65 - | | 1.49) | 0.9393 | |  |
| **145-3[GGG]** | | 45 | (11.6) | | 60 | (17.8) | **0.63** | **(0.40 -** | **0.97)** | **0.0378** |  | **0.52** | **(0.30 -** | | **0.89)** | **0.0163** | |  |
| 145-4[GAA] | | 6 | (1.6) | | 2 | (0.6) | 2.51 | (0.50 - | 12.60) | 0.2642 |  | 2.66 | (0.29 - | | 24.11) | 0.3843 | |  |
|  | |  |  | |  |  |  |  |  |  |  |  |  | |  |  | |  |
| 1234-1[AGCA] | | 147 | (37.9) | | 116 | (34.3) | Ref. |  |  |  |  | Ref. |  | |  |  | |  |
| 1234-2[AGCG] | | 116 | (29.9) | | 104 | (30.8) | 0.88 | (0.61 - | 1.26) | 0.4866 |  | 0.94 | (0.59 - | | 1.48) | 0.7763 | |  |
| 1234-3[ATCG] | | 56 | (14.4) | | 47 | (13.9) | 0.94 | (0.60 - | 1.49) | 0.7919 |  | 1.14 | (0.62 - | | 2.11) | 0.6731 | |  |
| 1234-4[GGTG] | | 33 | (8.5) | | 39 | (11.5) | 0.67 | (0.40 - | 1.13) | 0.1306 |  | 0.59 | (0.31 - | | 1.13) | 0.1105 | |  |
| **1234-5[GGCG]** | | 11 | (2.8) | | 20 | (5.9) | **0.43** | **(0.20 -** | **0.94)** | **0.0348** |  | **0.41** | **(0.17 -** | | **1.00)** | **0.0493** | |  |
| 1234-6[AGTG] | | 18 | (4.6) | | 9 | (2.7) | 1.58 | (0.68 - | 3.64) | 0.2850 |  | 0.97 | (0.35 - | | 2.74) | 0.9598 | |  |
| 1234-7[GGCA] | | 7 | (1.8) | | 3 | (0.9) | 1.84 | (0.47 - | 7.27) | 0.3842 |  | 1.54 | (0.28 - | | 8.36) | 0.6191 | |  |
|  | |  |  | |  |  |  |  |  |  |  |  |  | |  |  | |  |
| 1235-1[AGCA] | | 147 | (37.9) | | 118 | (34.9) | Ref. |  |  |  |  | Ref. |  | |  |  | |  |
| 1235-2[AGCG] | | 116 | (29.9) | | 102 | (30.2) | 0.91 | (0.64 - | 1.31) | 0.6196 |  | 0.99 | (0.63 - | | 1.58) | 0.9798 | |  |
| 1235-3[ATCG] | | 56 | (14.4) | | 47 | (13.9) | 0.96 | (0.61 - | 1.51) | 0.8486 |  | 1.17 | (0.64 - | | 2.16) | 0.6071 | |  |
| 1235-4[GGTG] | | 33 | (8.5) | | 39 | (11.5) | 0.68 | (0.40 - | 1.15) | 0.1472 |  | 0.61 | (0.32 - | | 1.16) | 0.1306 | |  |
| **1235-5[GGCG]** | | 11 | (2.8) | | 20 | (5.9) | **0.44** | **(0.20 -** | **0.96)** | **0.0386** |  | **0.42** | **(0.17 -** | | **1.03)** | **0.0566** | |  |
| 1235-6[AGTG] | | 18 | (4.6) | | 9 | (2.7) | 1.61 | (0.70 - | 3.70) | 0.2672 |  | 1.00 | (0.36 - | | 2.82) | 0.9972 | |  |
| 1235-7[GGCA] | | 7 | (1.8) | | 3 | (0.9) | 1.87 | (0.47 - | 7.40) | 0.3710 |  | 1.58 | (0.29 - | | 8.60) | 0.5961 | |  |
|  | |  |  | |  |  |  |  |  |  |  |  |  | |  |  | |  |
| 1245-1[AGAA] | | 147 | (37.9) | | 116 | (34.4) | Ref. |  |  |  |  | Ref. |  | |  |  | |  |
| 1245-2[AGGG] | | 134 | (34.5) | | 112 | (33.2) | 0.94 | (0.67 - | 1.34) | 0.7472 |  | 0.95 | (0.61 - | | 1.49) | 0.8235 | |  |
| 1245-3[ATGG] | | 56 | (14.4) | | 47 | (14.0) | 0.94 | (0.60 - | 1.49) | 0.7919 |  | 1.14 | (0.62 - | | 2.11) | 0.6720 | |  |
| **1245-4[GGGG]** | | 44 | (11.3) | | 59 | (17.5) | **0.59** | **(0.37 -** | **0.93)** | **0.0239** |  | **0.53** | **(0.30 -** | | **0.92)** | **0.0249** | |  |
| 1245-5[GGAA] | | 7 | (1.8) | | 3 | (0.9) | 1.84 | (0.47 - | 7.27) | 0.3842 |  | 1.54 | (0.28 - | | 8.37) | 0.6183 | |  |
|  | |  |  | |  |  |  |  |  |  |  |  |  | |  |  | |  |
| 1345-1[ACGG] | | 171 | (44.1) | | 149 | (44.2) | Ref. |  |  |  |  | Ref. |  | |  |  | |  |
| 1345-2[ACAA] | | 148 | (38.1) | | 117 | (34.7) | 1.10 | (0.80 - | 1.53) | 0.5599 |  | 0.98 | (0.64 - | | 1.50) | 0.9300 | |  |
| 1345-3[GTGG] | | 33 | (8.5) | | 39 | (11.6) | 0.74 | (0.44 - | 1.23) | 0.2442 |  | 0.59 | (0.31 - | | 1.10) | 0.0975 | |  |
| **1345-4[GCGG]** | | 12 | (3.1) | | 21 | (6.2) | **0.50** | **(0.24 -** | **1.05)** | **0.0657** |  | **0.40** | **(0.17 -** | | **0.95)** | **0.0366** | |  |
| 1345-5[ATGG] | | 18 | (4.6) | | 9 | (2.7) | 1.74 | (0.76 - | 4.00) | 0.1895 |  | 0.97 | (0.35 - | | 2.70) | 0.9462 | |  |
| 1345-6[GCAA] | | 6 | (1.6) | | 2 | (0.6) | 2.61 | (0.52 - | 13.15) | 0.2436 |  | 2.65 | (0.29 - | | 24.05) | 0.3865 | |  |
|  | |  |  | |  |  |  |  |  |  |  |  |  | |  |  | |  |
| 12345-1[AGCAA] | | 147 | (37.9) | | 116 | (34.3) | Ref. |  |  |  |  | Ref. |  | |  |  | |  |
| 12345-2[AGCGG] | | 116 | (29.9) | | 103 | (30.5) | 0.89 | (0.62 - | 1.27) | 0.5208 |  | 0.95 | (0.60 - | | 1.50) | 0.8205 | |  |
| 12345-3[ATCGG] | | 56 | (14.4) | | 47 | (13.9) | 0.94 | (0.60 - | 1.49) | 0.7919 |  | 1.14 | (0.62 - | | 2.10) | 0.6743 | |  |
| 12345-4[GGTGG] | | 33 | (8.5) | | 39 | (11.5) | 0.67 | (0.40 - | 1.13) | 0.1306 |  | 0.59 | (0.31 - | | 1.13) | 0.1108 | |  |
| **12345-5[GGCGG]** | | 11 | (2.8) | | 20 | (5.9) | **0.43** | **(0.20 -** | **0.94)** | **0.0348** |  | **0.41** | **(0.17 -** | | **1.00)** | **0.0489** | |  |
| 12345-6[AGTGG] | | 18 | (4.6) | | 9 | (2.7) | 1.58 | (0.68 - | 3.64) | 0.2849 |  | 0.97 | (0.35 - | | 2.74) | 0.9603 | |  |
| 12345-7[GGCAA] | | 7 | (1.8) | | 3 | (0.9) | 1.84 | (0.47 - | 7.28) | 0.3839 |  | 1.54 | (0.28 - | | 8.36) | 0.6190 | |  |
|  | |  |  | |  |  |  |  |  | |  |  |  |  | | | |  |
| **Haplotype* pair** | | **No. of  copies** |  | |  |  |  |  |  | |  |  |  |  | | | |  |
| 12-1 [AG] | | 0 | 13 | | (6.7) | 19 | (11.2) | Ref. |  |  |  |  | Ref. |  |  | |  | |
|  | | 1 or 2 | 181 | | (93.3) | 150 | (88.8) | 1.76 | (0.84 - | 3.69) | 0.1318 |  | 2.17 | (0.91 - | 5.14) | | 0.0797 | |
| 12-2 [GG] | | 0 | 145 | | (74.7) | 110 | (65.1) | Ref. |  |  |  |  | Ref. |  |  | |  | |
|  | | 1 or 2 | 49 | | (25.3) | 59 | (34.9) | 0.63 | (0.40 - | 0.99) | **0.0455** |  | 0.51 | (0.29 - | 0.90) | | 0.0199 | |
| 12-3 [AT] | | 0 | 143 | | (73.7) | 124 | (73.4) | Ref. |  |  |  |  | Ref. |  |  | |  | |
|  | | 1 or 2 | 51 | | (26.3) | 45 | (26.6) | 0.98 | (0.62 - | 1.57) | 0.9418 |  | 1.32 | (0.70 - | 2.47) | | 0.3871 | |
|  | |  |  | |  |  |  |  |  |  |  |  |  |  |  | |  | |
| 13-1 [AC] | | 0 | 7 | | (3.6) | 4 | (2.4) | Ref. |  |  |  |  | Ref. |  |  | |  | |
|  | | 1 or 2 | 187 | | (96.4) | 165 | (97.6) | 0.65 | (0.19 - | 2.25) | 0.4944 |  | 1.10 | (0.26 - | 4.69) | | 0.9025 | |
| 13-2 [GT] | | 0 | 162 | | (83.5) | 132 | (78.1) | Ref. |  |  |  |  | Ref. |  |  | |  | |
|  | | 1 or 2 | 32 | | (16.5) | 37 | (21.9) | 0.70 | (0.42 - | 1.19) | 0.1922 |  | 0.61 | (0.32 - | 1.18) | | 0.1414 | |
| 13-3 [GC] | | 0 | 176 | | (90.7) | 146 | (86.4) | Ref. |  |  |  |  | Ref. |  |  | |  | |
|  | | 1 or 2 | 18 | | (9.3) | 23 | (13.6) | 0.65 | (0.34 - | 1.25) | 0.1956 |  | 0.56 | (0.26 - | 1.21) | | 0.1400 | |
| 13-4 [AT] | | 0 | 177 | | (91.2) | 161 | (95.3) | Ref. |  |  |  |  | Ref. |  |  | |  | |
|  | | 1 or 2 | 17 | | (8.8) | 8 | (4.7) | 1.93 | (0.81 - | 4.60) | 0.1364 |  | 1.18 | (0.41 - | 3.41) | | 0.7618 | |
|  | |  |  | |  |  |  |  |  |  |  |  |  |  |  | |  | |
| 14-1 [AG] | | 0 | 57 | | (29.4) | 48 | (28.4) | Ref. |  |  |  |  | Ref. |  |  | |  | |
|  | | 1 or 2 | 137 | | (70.6) | 121 | (71.6) | 0.95 | (0.60 - | 1.50) | 0.8375 |  | 0.97 | (0.55 - | 1.72) | | 0.9152 | |
| 14-2 [AA] | | 0 | 77 | | (39.7) | 77 | (45.6) | Ref. |  |  |  |  | Ref. |  |  | |  | |
|  | | 1 or 2 | 117 | | (60.3) | 92 | (54.4) | 1.27 | (0.84 - | 1.93) | 0.2592 |  | 1.22 | (0.72 - | 2.07) | | 0.4686 | |
| 14-3 [GG] | | 0 | 153 | | (78.9) | 112 | (66.3) | Ref. |  |  |  |  | Ref. |  |  | |  | |
|  | | 1 or 2 | 41 | | (21.1) | 57 | (33.7) | 0.53 | (0.33 - | 0.84) | **0.0074** |  | 0.43 | (0.24 - | 0.77) | | **0.0047** | |
| 14-4 [GA] | | 0 | 189 | | (97.4) | 167 | (98.8) | Ref. |  |  |  |  | Ref. |  |  | |  | |
|  | | 1 or 2 | 5 | | (2.6) | 2 | (1.2) | 2.21 | (0.42 - | 11.53) | 0.3477 |  | 2.19 | (0.23 - | 21.07) | | 0.4982 | |
|  | |  |  | |  |  |  |  |  |  |  |  |  |  |  | |  | |
| 15-1 [AG] | | 0 | 48 | | (24.7) | 49 | (29.0) | Ref. |  |  |  |  | Ref. |  |  | |  | |
|  | | 1 or 2 | 146 | | (75.3) | 120 | (71.0) | 1.24 | (0.78 - | 1.98) | 0.3615 |  | 1.29 | (0.72 - | 2.32) | | 0.3921 | |
| 15-2 [AA] | | 0 | 72 | | (37.1) | 75 | (44.4) | Ref. |  |  |  |  | Ref. |  |  | |  | |
|  | | 1 or 2 | 122 | | (62.9) | 94 | (55.6) | 1.35 | (0.89 - | 2.06) | 0.1600 |  | 1.32 | (0.77 - | 2.25) | | 0.3113 | |
| 15-3 [GG] | | 0 | 150 | | (77.3) | 112 | (66.3) | Ref. |  |  |  |  | Ref. |  |  | |  | |
|  | | 1 or 2 | 44 | | (22.7) | 57 | (33.7) | 0.58 | (0.36 - | 0.92) | **0.0197** |  | 0.47 | (0.27 - | 0.84) | | **0.0104** | |
| 15-4 [GA] | | 0 | 188 | | (96.9) | 167 | (98.8) | Ref. |  |  |  |  | Ref. |  |  | |  | |
|  | | 1 or 2 | 6 | | (3.1) | 2 | (1.2) | 2.66 | (0.53 - | 13.38) | 0.2339 |  | 3.03 | (0.33 - | 27.44) | | 0.3242 | |
|  | |  |  | |  |  |  |  |  |  |  |  |  |  |  | |  | |
| 123-1 [AGC] | | 0 | 20 | | (10.3) | 20 | (11.8) | Ref. |  |  |  |  | Ref. |  |  | |  | |
|  | | 1 or 2 | 174 | | (89.7) | 149 | (88.2) | 1.17 | (0.61 - | 2.25) | 0.6428 |  | 1.67 | (0.75 - | 3.70) | | 0.2078 | |
| 123-2 [ATC] | | 0 | 143 | | (73.7) | 124 | (73.4) | Ref. |  |  |  |  | Ref. |  |  | |  | |
|  | | 1 or 2 | 51 | | (26.3) | 45 | (26.6) | 0.98 | (0.62 - | 1.57) | 0.9418 |  | 1.32 | (0.70 - | 2.47) | | 0.3871 | |
| 123-3 [GGT] | | 0 | 162 | | (83.5) | 132 | (78.1) | Ref. |  |  |  |  | Ref. |  |  | |  | |
|  | | 1 or 2 | 32 | | (16.5) | 37 | (21.9) | 0.70 | (0.42 - | 1.19) | 0.1922 |  | 0.61 | (0.32 - | 1.18) | | 0.1414 | |
| 123-4 [GGC] | | 0 | 176 | | (90.7) | 146 | (86.4) | Ref. |  |  |  |  | Ref. |  |  | |  | |
|  | | 1 or 2 | 18 | | (9.3) | 23 | (13.6) | 0.65 | (0.34 - | 1.25) | 0.1956 |  | 0.56 | (0.26 - | 1.21) | | 0.1400 | |
| 123-5 [AGT] | | 0 | 177 | | (91.2) | 161 | (95.3) | Ref. |  |  |  |  | Ref. |  |  | |  | |
|  | | 1 or 2 | 17 | | (8.8) | 8 | (4.7) | 1.93 | (0.81 - | 4.60) | 0.1364 |  | 1.18 | (0.41 - | 3.41) | | 0.7618 | |
|  | |  |  | |  |  |  |  |  |  |  |  |  |  |  | |  | |
| 124-1 [AGA] | | 0 | 71 | | (36.6) | 77 | (45.6) | Ref. |  |  |  |  | Ref. |  |  | |  | |
|  | | 1 or 2 | 123 | | (63.4) | 92 | (54.4) | 1.45 | (0.95 - | 2.21) | 0.0835 |  | 1.41 | (0.83 - | 2.39) | | 0.2099 | |
| 124-2 [AGG] | | 0 | 83 | | (42.8) | 77 | (45.6) | Ref. |  |  |  |  | Ref. |  |  | |  | |
|  | | 1 or 2 | 111 | | (57.2) | 92 | (54.4) | 1.12 | (0.74 - | 1.70) | 0.5948 |  | 1.15 | (0.68 - | 1.96) | | 0.6063 | |
| 124-3 [ATG] | | 0 | 143 | | (73.7) | 124 | (73.4) | Ref. |  |  |  |  | Ref. |  |  | |  | |
|  | | 1 or 2 | 51 | | (26.3) | 45 | (26.6) | 0.98 | (0.62 - | 1.57) | 0.9418 |  | 1.32 | (0.70 - | 2.47) | | 0.3871 | |
| 124-4 [GGG] | | 0 | 151 | | (77.8) | 113 | (66.9) | Ref. |  |  |  |  | Ref. |  |  | |  | |
|  | | 1 or 2 | 43 | | (22.2) | 56 | (33.1) | 0.57 | (0.36 - | 0.92) | **0.0199** |  | 0.48 | (0.27 - | 0.86) | | **0.0132** | |
| 124-5 [GGA] | | 0 | 187 | | (96.4) | 166 | (98.2) | Ref. |  |  |  |  | Ref. |  |  | |  | |
|  | | 1 or 2 | 7 | | (3.6) | 3 | (1.8) | 2.07 | (0.53 - | 8.14) | 0.2972 |  | 1.73 | (0.32 - | 9.35) | | 0.5231 | |
|  | |  |  | |  |  |  |  |  |  |  |  |  |  |  | |  | |
| 125-1 [AGA] | | 0 | 73 | | (37.6) | 76 | (45.0) | Ref. |  |  |  |  | Ref. |  |  | |  | |
|  | | 1 or 2 | 121 | | (62.4) | 93 | (55.0) | 1.35 | (0.89 - | 2.06) | 0.1565 |  | 1.35 | (0.79 - | 2.30) | | 0.2705 | |
| 125-2 [AGG] | | 0 | 81 | | (41.8) | 77 | (45.6) | Ref. |  |  |  |  | Ref. |  |  | |  | |
|  | | 1 or 2 | 113 | | (58.2) | 92 | (54.4) | 1.17 | (0.77 - | 1.77) | 0.4654 |  | 1.16 | (0.68 - | 1.98) | | 0.5800 | |
| 125-3 [ATG] | | 0 | 143 | | (73.7) | 124 | (73.4) | Ref. |  |  |  |  | Ref. |  |  | |  | |
|  | | 1 or 2 | 51 | | (26.3) | 45 | (26.6) | 0.98 | (0.62 - | 1.57) | 0.9418 |  | 1.32 | (0.70 - | 2.47) | | 0.3871 | |
| 125-4 [GGG] | | 0 | 151 | | (77.8) | 113 | (66.9) | Ref. |  |  |  |  | Ref. |  |  | |  | |
|  | | 1 or 2 | 43 | | (22.2) | 56 | (33.1) | 0.57 | (0.36 - | 0.92) | **0.0199** |  | 0.48 | (0.27 - | 0.86) | | **0.0132** | |
| 125-5 [GGA] | | 0 | 187 | | (96.4) | 166 | (98.2) | Ref. |  |  |  |  | Ref. |  |  | |  | |
|  | | 1 or 2 | 7 | | (3.6) | 3 | (1.8) | 2.07 | (0.53 - | 8.14) | 0.2972 |  | 1.73 | (0.32 - | 9.35) | | 0.5231 | |
|  | |  |  | |  |  |  |  |  |  |  |  |  |  |  | |  | |
| 134-1 [ACG] | | 0 | 61 | | (31.4) | 51 | (30.2) | Ref. |  |  |  |  | Ref. |  |  | |  | |
|  | | 1 or 2 | 133 | | (68.6) | 118 | (69.8) | 0.94 | (0.60 - | 1.47) | 0.7947 |  | 1.12 | (0.64 - | 1.97) | | 0.6964 | |
| 134-2 [ACA] | | 0 | 70 | | (36.1) | 77 | (45.6) | Ref. |  |  |  |  | Ref. |  |  | |  | |
|  | | 1 or 2 | 124 | | (63.9) | 92 | (54.4) | 1.48 | (0.97 - | 2.26) | 0.0670 |  | 1.42 | (0.84 - | 2.43) | | 0.1929 | |
| 134-3 [GTG] | | 0 | 162 | | (83.5) | 132 | (78.1) | Ref. |  |  |  |  | Ref. |  |  | |  | |
|  | | 1 or 2 | 32 | | (16.5) | 37 | (21.9) | 0.70 | (0.42 - | 1.19) | 0.1922 |  | 0.61 | (0.32 - | 1.18) | | 0.1414 | |
| 134-4 [GCG] | | 0 | 182 | | (93.8) | 148 | (87.6) | Ref. |  |  |  |  | Ref. |  |  | |  | |
|  | | 1 or 2 | 12 | | (6.2) | 21 | (12.4) | 0.46 | (0.22 - | 0.98) | **0.0428** |  | 0.40 | (0.17 - | 0.95) | | **0.0379** | |
| 134-5 [ATG] | | 0 | 177 | | (91.2) | 161 | (95.3) | Ref. |  |  |  |  | Ref. |  |  | |  | |
|  | | 1 or 2 | 17 | | (8.8) | 8 | (4.7) | 1.93 | (0.81 - | 4.60) | 0.1364 |  | 1.18 | (0.41 - | 3.41) | | 0.7618 | |
| 134-6 [GCA] | | 0 | 188 | | (96.9) | 167 | (98.8) | Ref. |  |  |  |  | Ref. |  |  | |  | |
|  | | 1 or 2 | 6 | | (3.1) | 2 | (1.2) | 2.66 | (0.53 - | 13.38) | 0.2339 |  | 3.03 | (0.33 - | 27.44) | | 0.3242 | |
|  | |  |  | |  |  |  |  |  |  |  |  |  |  |  | |  | |
| 135-1 [ACG] | | 0 | 59 | | (30.4) | 52 | (30.8) | Ref. |  |  |  |  | Ref. |  |  | |  | |
|  | | 1 or 2 | 135 | | (69.6) | 117 | (69.2) | 1.02 | (0.65 - | 1.59) | 0.9413 |  | 1.18 | (0.67 - | 2.08) | | 0.5638 | |
| 135-2 [ACA] | | 0 | 72 | | (37.1) | 75 | (44.4) | Ref. |  |  |  |  | Ref. |  |  | |  | |
|  | | 1 or 2 | 122 | | (62.9) | 94 | (55.6) | 1.35 | (0.89 - | 2.06) | 0.1600 |  | 1.32 | (0.77 - | 2.25) | | 0.3113 | |
| 135-3 [GTG] | | 0 | 162 | | (83.5) | 132 | (78.1) | Ref. |  |  |  |  | Ref. |  |  | |  | |
|  | | 1 or 2 | 32 | | (16.5) | 37 | (21.9) | 0.70 | (0.42 - | 1.19) | 0.1922 |  | 0.61 | (0.32 - | 1.18) | | 0.1414 | |
| 135-4 [GCG] | | 0 | 182 | | (93.8) | 148 | (87.6) | Ref. |  |  |  |  | Ref. |  |  | |  | |
|  | | 1 or 2 | 12 | | (6.2) | 21 | (12.4) | 0.46 | (0.22 - | 0.98) | **0.0428** |  | 0.40 | (0.17 - | 0.95) | | **0.0379** | |
| 135-5 [ATG] | | 0 | 177 | | (91.2) | 161 | (95.3) | Ref. |  |  |  |  | Ref. |  |  | |  | |
|  | | 1 or 2 | 17 | | (8.8) | 8 | (4.7) | 1.93 | (0.81 - | 4.60) | 0.1364 |  | 1.18 | (0.41 - | 3.41) | | 0.7618 | |
| 135-6 [GCA] | | 0 | 188 | | (96.9) | 167 | (98.8) | Ref. |  |  |  |  | Ref. |  |  | |  | |
|  | | 1 or 2 | 6 | | (3.1) | 2 | (1.2) | 2.66 | (0.53 - | 13.38) | 0.2339 |  | 3.03 | (0.33 - | 27.44) | | 0.3242 | |
|  | |  |  | |  |  |  |  |  |  |  |  |  |  |  | |  | |
| 145-1 [AGG] | | 0 | 50 | | (25.8) | 49 | (29.0) | Ref. |  |  |  |  | Ref. |  |  | |  | |
|  | | 1 or 2 | 144 | | (74.2) | 120 | (71.0) | 1.18 | (0.74 - | 1.87) | 0.4921 |  | 1.27 | (0.71 - | 2.28) | | 0.4166 | |
| 145-2 [AAA] | | 0 | 70 | | (36.1) | 76 | (45.0) | Ref. |  |  |  |  | Ref. |  |  | |  | |
|  | | 1 or 2 | 124 | | (63.9) | 93 | (55.0) | 1.45 | (0.95 - | 2.21) | 0.0855 |  | 1.37 | (0.81 - | 2.34) | | 0.2440 | |
| 145-3 [GGG] | | 0 | 150 | | (77.3) | 112 | (66.3) | Ref. |  |  |  |  | Ref. |  |  | |  | |
|  | | 1 or 2 | 44 | | (22.7) | 57 | (33.7) | 0.58 | (0.36 - | 0.92) | **0.0197** |  | 0.47 | (0.27 - | 0.84) | | **0.0104** | |
| 145-4 [GAA] | | 0 | 188 | | (96.9) | 167 | (98.8) | Ref. |  |  |  |  | Ref. |  |  | |  | |
|  | | 1 or 2 | 6 | | (3.1) | 2 | (1.2) | 2.66 | (0.53 - | 13.38) | 0.2339 |  | 3.03 | (0.33 - | 27.44) | | 0.3242 | |
| 145-5 [AGA] | | 0 | 194 | | (100.0) | 168 | (99.4) | Ref. |  |  |  |  | Ref. |  |  | |  | |
|  | | 1 or 2 | 0 | | (0.0) | 1 | (0.6) | - |  |  |  |  | - |  |  | |  | |
|  | |  |  | |  |  |  |  |  |  |  |  |  |  |  | |  | |
| 1234-1 [AGCA] | | 0 | 71 | | (36.6) | 77 | (45.6) | Ref. |  |  |  |  | Ref. |  |  | |  | |
|  | | 1 or 2 | 123 | | (63.4) | 92 | (54.4) | 1.45 | (0.95 - | 2.21) | 0.0835 |  | 1.41 | (0.83 - | 2.39) | | 0.2099 | |
| 1234-2 [AGCG] | | 0 | 96 | | (49.5) | 80 | (47.3) | Ref. |  |  |  |  | Ref. |  |  | |  | |
|  | | 1 or 2 | 98 | | (50.5) | 89 | (52.7) | 0.92 | (0.61 - | 1.39) | 0.6831 |  | 1.04 | (0.61 - | 1.77) | | 0.8724 | |
| 1234-3 [ATCG] | | 0 | 143 | | (73.7) | 124 | (73.4) | Ref. |  |  |  |  | Ref. |  |  | |  | |
|  | | 1 or 2 | 51 | | (26.3) | 45 | (26.6) | 0.98 | (0.62 - | 1.57) | 0.9418 |  | 1.32 | (0.70 - | 2.47) | | 0.3871 | |
| 1234-4 [GGTG] | | 0 | 162 | | (83.5) | 132 | (78.1) | Ref. |  |  |  |  | Ref. |  |  | |  | |
|  | | 1 or 2 | 32 | | (16.5) | 37 | (21.9) | 0.70 | (0.42 - | 1.19) | 0.1922 |  | 0.61 | (0.32 - | 1.18) | | 0.1414 | |
| 1234-5 [GGCG] | | 0 | 183 | | (94.3) | 149 | (88.2) | Ref. |  |  |  |  | Ref. |  |  | |  | |
|  | | 1 or 2 | 11 | | (5.7) | 20 | (11.8) | 0.45 | (0.21 - | 0.96) | **0.0400** |  | 0.41 | (0.17 - | 0.99) | | **0.0472** | |
| 1234-6 [AGTG] | | 0 | 177 | | (91.2) | 161 | (95.3) | Ref. |  |  |  |  | Ref. |  |  | |  | |
|  | | 1 or 2 | 17 | | (8.8) | 8 | (4.7) | 1.93 | (0.81 - | 4.60) | 0.1364 |  | 1.18 | (0.41 - | 3.41) | | 0.7618 | |
| 1234-7 [GGCA] | | 0 | 187 | | (96.4) | 166 | (98.2) | Ref. |  |  |  |  | Ref. |  |  | |  | |
|  | | 1 or 2 | 7 | | (3.6) | 3 | (1.8) | 2.07 | (0.53 - | 8.14) | 0.2972 |  | 1.73 | (0.32 - | 9.35) | | 0.5231 | |
|  | |  |  | |  |  |  |  |  |  |  |  |  |  |  | |  | |
| 1235-1 [AGCA] | | 0 | 71 | | (36.6) | 75 | (44.4) | Ref. |  |  |  |  | Ref. |  |  | |  | |
|  | | 1 or 2 | 123 | | (63.4) | 94 | (55.6) | 1.38 | (0.91 - | 2.11) | 0.1320 |  | 1.30 | (0.76 - | 2.22) | | 0.3331 | |
| 1235-2 [AGCG] | | 0 | 96 | | (49.5) | 81 | (47.9) | Ref. |  |  |  |  | Ref. |  |  | |  | |
|  | | 1 or 2 | 98 | | (50.5) | 88 | (52.1) | 0.94 | (0.62 - | 1.42) | 0.7675 |  | 1.09 | (0.64 - | 1.86) | | 0.7393 | |
| 1235-3 [ATCG] | | 0 | 143 | | (73.7) | 124 | (73.4) | Ref. |  |  |  |  | Ref. |  |  | |  | |
|  | | 1 or 2 | 51 | | (26.3) | 45 | (26.6) | 0.98 | (0.62 - | 1.57) | 0.9418 |  | 1.32 | (0.70 - | 2.47) | | 0.3871 | |
| 1235-4 [GGTG] | | 0 | 162 | | (83.5) | 132 | (78.1) | Ref. |  |  |  |  | Ref. |  |  | |  | |
|  | | 1 or 2 | 32 | | (16.5) | 37 | (21.9) | 0.70 | (0.42 - | 1.19) | 0.1922 |  | 0.61 | (0.32 - | 1.18) | | 0.1414 | |
| 1235-5 [GGCG] | | 0 | 183 | | (94.3) | 149 | (88.2) | Ref. |  |  |  |  | Ref. |  |  | |  | |
|  | | 1 or 2 | 11 | | (5.7) | 20 | (11.8) | 0.45 | (0.21 - | 0.96) | **0.0400** |  | 0.41 | (0.17 - | 0.99) | | **0.0472** | |
| 1235-6 [AGTG] | | 0 | 177 | | (91.2) | 161 | (95.3) | Ref. |  |  |  |  | Ref. |  |  | |  | |
|  | | 1 or 2 | 17 | | (8.8) | 8 | (4.7) | 1.93 | (0.81 - | 4.60) | 0.1364 |  | 1.18 | (0.41 - | 3.41) | | 0.7618 | |
| 1235-7 [GGCA] | | 0 | 187 | | (96.4) | 166 | (98.2) | Ref. |  |  |  |  | Ref. |  |  | |  | |
|  | | 1 or 2 | 7 | | (3.6) | 3 | (1.8) | 2.07 | (0.53 - | 8.14) | 0.2972 |  | 1.73 | (0.32 - | 9.35) | | 0.5231 | |
|  | |  |  | |  |  |  |  |  |  |  |  |  |  |  | |  | |
| 1245-1 [AGAA] | | 0 | 71 | | (36.6) | 77 | (45.6) | Ref. |  |  |  |  | Ref. |  |  | |  | |
|  | | 1 or 2 | 123 | | (63.4) | 92 | (54.4) | 1.45 | (0.95 - | 2.21) | 0.0835 |  | 1.41 | (0.83 - | 2.39) | | 0.2099 | |
| 1245-2 [AGGG] | | 0 | 83 | | (42.8) | 77 | (45.6) | Ref. |  |  |  |  | Ref. |  |  | |  | |
|  | | 1 or 2 | 111 | | (57.2) | 92 | (54.4) | 1.12 | (0.74 - | 1.70) | 0.5948 |  | 1.15 | (0.68 - | 1.96) | | 0.6063 | |
| 1245-3 [ATGG] | | 0 | 143 | | (73.7) | 124 | (73.4) | Ref. |  |  |  |  | Ref. |  |  | |  | |
|  | | 1 or 2 | 51 | | (26.3) | 45 | (26.6) | 0.98 | (0.62 - | 1.57) | 0.9418 |  | 1.32 | (0.70 - | 2.47) | | 0.3871 | |
| 1245-4 [GGGG] | | 0 | 151 | | (77.8) | 113 | (66.9) | Ref. |  |  |  |  | Ref. |  |  | |  | |
|  | | 1 or 2 | 43 | | (22.2) | 56 | (33.1) | 0.57 | (0.36 - | 0.92) | **0.0199** |  | 0.48 | (0.27 - | 0.86) | | **0.0132** | |
| 1245-5 [GGAA] | | 0 | 187 | | (96.4) | 166 | (98.2) | Ref. |  |  |  |  | Ref. |  |  | |  | |
|  | | 1 or 2 | 7 | | (3.6) | 3 | (1.8) | 2.07 | (0.53 - | 8.14) | 0.2972 |  | 1.73 | (0.32 - | 9.35) | | 0.5231 | |
| 1245-6 [AGGA] | | 0 | 194 | | (100.0) | 168 | (99.4) | Ref. |  |  |  |  | Ref. |  |  | |  | |
|  | | 1 or 2 | 0 | | (0.0) | 1 | (0.6) | - |  |  |  |  | - |  |  | |  | |
|  | |  |  | |  |  |  |  |  |  |  |  |  |  |  | |  | |
| 1345-1 [ACGG] | | 0 | 61 | | (31.4) | 52 | (30.8) | Ref. |  |  |  |  | Ref. |  |  | |  | |
|  | | 1 or 2 | 133 | | (68.6) | 117 | (69.2) | 0.97 | (0.62 - | 1.51) | 0.8900 |  | 1.17 | (0.66 - | 2.05) | | 0.5918 | |
| 1345-2 [ACAA] | | 0 | 70 | | (36.1) | 76 | (45.0) | Ref. |  |  |  |  | Ref. |  |  | |  | |
|  | | 1 or 2 | 124 | | (63.9) | 93 | (55.0) | 1.45 | (0.95 - | 2.21) | 0.0855 |  | 1.37 | (0.81 - | 2.34) | | 0.2440 | |
| 1345-3 [GTGG] | | 0 | 162 | | (83.5) | 132 | (78.1) | Ref. |  |  |  |  | Ref. |  |  | |  | |
|  | | 1 or 2 | 32 | | (16.5) | 37 | (21.9) | 0.70 | (0.42 - | 1.19) | 0.1922 |  | 0.61 | (0.32 - | 1.18) | | 0.1414 | |
| 1345-4 [GCGG] | | 0 | 182 | | (93.8) | 148 | (87.6) | Ref. |  |  |  |  | Ref. |  |  | |  | |
|  | | 1 or 2 | 12 | | (6.2) | 21 | (12.4) | 0.46 | (0.22 - | 0.98) | **0.0428** |  | 0.40 | (0.17 - | 0.95) | | **0.0379** | |
| 1345-5 [ATGG] | | 0 | 177 | | (91.2) | 161 | (95.3) | Ref. |  |  |  |  | Ref. |  |  | |  | |
|  | | 1 or 2 | 17 | | (8.8) | 8 | (4.7) | 1.93 | (0.81 - | 4.60) | 0.1364 |  | 1.18 | (0.41 - | 3.41) | | 0.7618 | |
| 1345-6 [GCAA] | | 0 | 188 | | (96.9) | 167 | (98.8) | Ref. |  |  |  |  | Ref. |  |  | |  | |
|  | | 1 or 2 | 6 | | (3.1) | 2 | (1.2) | 2.66 | (0.53 - | 13.38) | 0.2339 |  | 3.03 | (0.33 - | 27.44) | | 0.3242 | |
| 1345-7 [ACGA] | | 0 | 194 | | (100.0) | 168 | (99.4) | Ref. |  |  |  |  | Ref. |  |  | |  | |
|  | | 1 or 2 | 0 | | (0.0) | 1 | (0.6) | - |  |  |  |  | - |  |  | |  | |
|  | |  |  | |  |  |  |  |  |  |  |  |  |  |  | |  | |
| 12345-1 [AGCAA] | | 0 | 71 | | (36.6) | 77 | (45.6) | Ref. |  |  |  |  | Ref. |  |  | |  | |
|  | | 1 or 2 | 123 | | (63.4) | 92 | (54.4) | 1.45 | (0.95 - | 2.21) | 0.0835 |  | 1.41 | (0.83 - | 2.39) | | 0.2099 | |
| 12345-2 [AGCGG] | | 0 | 96 | | (49.5) | 80 | (47.3) | Ref. |  |  |  |  | Ref. |  |  | |  | |
|  | | 1 or 2 | 98 | | (50.5) | 89 | (52.7) | 0.92 | (0.61 - | 1.39) | 0.6831 |  | 1.04 | (0.61 - | 1.77) | | 0.8724 | |
| 12345-3 [ATCGG] | | 0 | 143 | | (73.7) | 124 | (73.4) | Ref. |  |  |  |  | Ref. |  |  | |  | |
|  | | 1 or 2 | 51 | | (26.3) | 45 | (26.6) | 0.98 | (0.62 - | 1.57) | 0.9418 |  | 1.32 | (0.70 - | 2.47) | | 0.3871 | |
| 12345-4 [GGTGG] | | 0 | 162 | | (83.5) | 132 | (78.1) | Ref. |  |  |  |  | Ref. |  |  | |  | |
|  | | 1 or 2 | 32 | | (16.5) | 37 | (21.9) | 0.70 | (0.42 - | 1.19) | 0.1922 |  | 0.61 | (0.32 - | 1.18) | | 0.1414 | |
| 12345-5 [GGCGG] | | 0 | 183 | | (94.3) | 149 | (88.2) | Ref. |  |  |  |  | Ref. |  |  | |  | |
|  | | 1 or 2 | 11 | | (5.7) | 20 | (11.8) | 0.45 | (0.21 - | 0.96) | **0.0400** |  | 0.41 | (0.17 - | 0.99) | | **0.0472** | |
| 12345-6 [AGTGG] | | 0 | 177 | | (91.2) | 161 | (95.3) | Ref. |  |  |  |  | Ref. |  |  | |  | |
|  | | 1 or 2 | 17 | | (8.8) | 8 | (4.7) | 1.93 | (0.81 - | 4.60) | 0.1364 |  | 1.18 | (0.41 - | 3.41) | | 0.7618 | |
| 12345-7 [GGCAA] | | 0 | 187 | | (96.4) | 166 | (98.2) | Ref. |  |  |  |  | Ref. |  |  | |  | |
|  | | 1 or 2 | 7 | | (3.6) | 3 | (1.8) | 2.07 | (0.53 - | 8.14) | 0.2972 |  | 1.73 | (0.32 - | 9.35) | | 0.5231 | |
| 12345-8 [AGCGA] | | 0 | 194 | | (100.0) | 168 | (99.4) | Ref. |  |  |  |  | Ref. |  |  | |  | |
|  | | 1 or 2 | 0 | | (0.0) | 1 | (0.6) | - |  |  |  |  | - |  |  | |  | |

OR, odds ratio; CI, confidence interval; AMACR, (R)-alpha-methyl-CoA racemase

***** 1, rs2278008; 2, rs34677; 3, rs2287939; 4, rs10941112; 5, rs3195676

^a^ Adjusted for age, smoking quantity, alcohol drinking and family history of prostate cancer

**
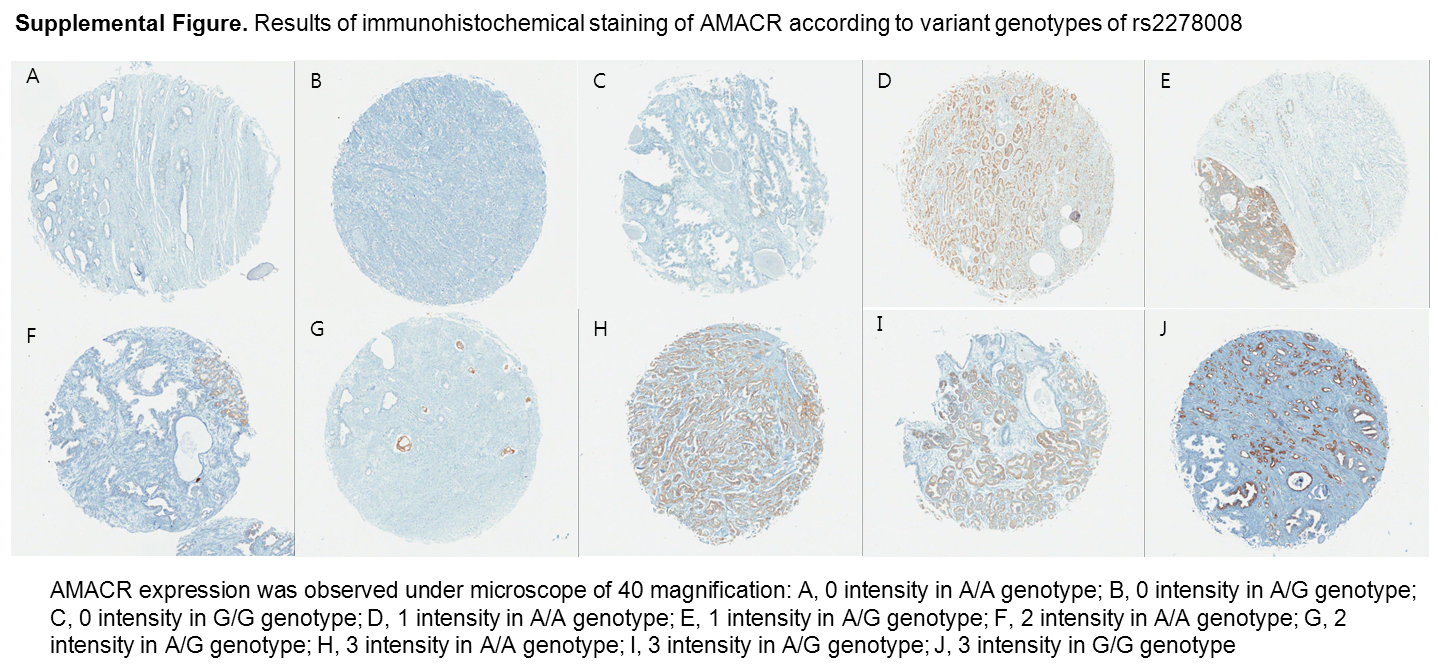
**
